# Patient-generated health data and electronic health record integration: a scoping review

**DOI:** 10.1093/jamiaopen/ooaa052

**Published:** 2020-12-05

**Authors:** Victoria L Tiase, William Hull, Mary M McFarland, Katherine A Sward, Guilherme Del Fiol, Catherine Staes, Charlene Weir, Mollie R Cummins

**Affiliations:** 1 University of Utah, College of Nursing, The Value Institute, NewYork-Presbyterian Hospital, New York, New York, USA; 2 University of Utah, College of Nursing, Salt Lake City, Utah, USA; 3 University of Utah, Eccles Health Sciences Library, Salt Lake City, Utah, USA; 4 Department of Biomedical Informatics, University of Utah, Salt Lake City, Utah, USA

**Keywords:** patient-generated health data, integration, precision medicine, consumer health informatics, interoperability

## Abstract

**Objectives:**

Patient-generated health data (PGHD) are clinically relevant data captured by patients outside of the traditional care setting. Clinical use of PGHD has emerged as an essential issue. This study explored the evidence to determine the extent of and describe the characteristics of PGHD integration into electronic health records (EHRs).

**Methods:**

In August 2019, we conducted a systematic scoping review. We included studies with complete, partial, or in-progress PGHD and EHR integration within a clinical setting. The retrieved articles were screened for eligibility by 2 researchers, and data from eligible articles were abstracted, coded, and analyzed.

**Results:**

A total of 19 studies met inclusion criteria after screening 9463 abstracts. Most of the study designs were pilots and all were published between 2013 and 2019. Types of PGHD were biometric and patient activity (57.9%), questionnaires and surveys (36.8%), and health history (5.3%). Diabetes was the most common patient condition (42.1%) for PGHD collection. Active integration (57.9%) was slightly more common than passive integration (31.6%). We categorized emergent themes into the 3 steps of PGHD flow. Themes emerged concerning resource requirements, data delivery to the EHR, and preferences for review.

**Discussion:**

PGHD integration into EHRs appears to be at an early stage. PGHD have the potential to close health care gaps and support personalized medicine. Efforts are needed to understand how to optimize PGHD integration into EHRs considering resources, standards for EHR delivery, and clinical workflows.

## INTRODUCTION

**LAY ABSTRACT**

Patient-generated health data (PGHD) are data captured by patients during their everyday lives and outside of the clinic or hospital. Introducing PGHD into the clinical setting so that clinicians can make use of these data is an essential issue. This study explored the evidence to determine the extent of and describe the characteristics of PGHD integration into electronic health records (EHRs). In August 2019, we conducted a systematic scoping review. The retrieved articles were screened for eligibility by 2 researchers, and data from eligible articles were abstracted, coded, and analyzed. A total of 19 studies met inclusion criteria after screening 9463 abstracts. We classified the types of PGHD as biometric and patient activity, questionnaires and surveys, and health history. Diabetes was the most common patient condition for PGHD collection. We categorized emergent themes and found that PGHD integration into EHRs appears to be at an early stage. PGHD have the potential to close health care gaps and support personalized medicine. Efforts are needed to understand how to optimize PGHD integration into EHRs considering resources, standards for EHR delivery, and clinical workflows.

Precision health initiatives are focused on predicting, preventing, and curing disease using technology and targeted programs. The *All of Us* Research Program, for example, will collect data from 1 million volunteers and examine the effects of differences in biological makeup, lifestyle, and environment.^1^ Other precision medicine programs are striving to improve cancer outcomes through tailored treatments, such as by analyzing patient data and then matching patients with treatments.^2^ Trends in care management are focused on using social determinants of health to inform patient care.^3^ Ultimately, these personalized efforts rely on patient-generated health data (PGHD); data relevant to health that were collected from or recorded by patients in their day to day lives.

With the proliferation of lower-cost wearables and mobile health technologies, patients are generating an abundance of data. PGHD encompass physiological data and lifestyle factors such as diet, fitness, and sleep.^4^ PGHD are collected through manual data entry, physiological monitoring, and environmental and biometric sensors to perform self-tracking between visits. PGHD collection empowers, engages, and encourages greater self-awareness in managing health.^5^ When shared with providers, PGHD can augment communication between patients and providers to facilitate shared decision-making and care planning.^6^

Federal and industry initiatives emphasize seamless sharing of patient data for clinical care. The 21st Century Cures Act, signed into law in 2016, includes provisions on data sharing and interoperability to encourage the access, exchange or use of electronic health data.^7^ A proposed rule from the Centers for Medicare and Medicaid Services requires healthcare facilities to implement technologies that support open application programming interfaces (APIs) that allow real-time bidirectional data exchange between patients and providers.^8^ The Office of the National Coordinator for Health IT (ONC) proposed an approach to facilitate the communication exchange in a standard, secure way without special effort on the part of the user.^9^ These regulations are expected to accelerate the development of mobile health (mHealth) technologies that are compliant with PGHD interoperability standards.^10^

However, there are barriers to integrating PGHD into clinical care. Despite advances in electronic health record (EHR) capabilities, clinicians typically rely on patient recall and manually enter between-visit data, resulting in an incomplete picture of the patient’s experience. PGHD are generally collected in a separate platform, requiring providers to access dashboards or reports outside of the EHR. The need to access disparate data sources, remember additional passwords, and assimilate new data outside of care workflows contributes to clinicians’ information overload.^11^ On the other hand, successful integration of PGHD may provide efficiencies and potentially impact patient outcomes.^12^ Best practices for successful integrations are needed.^13^

Currently, there are few examples of large scale PGHD use in the clinical setting. Previous research identified that many PGHD efforts focus on small pilot efforts in research settings.^14^ A systematic review of symptom-related PGHD found just 21 studies and reported minimal integration.^15^ Another recent study examined patient and provider perspectives regarding PGHD and found electronic integration of PGHD into existing systems to be an important but largely unmet need.^16^ A study that characterized approaches to PGHD use concluded that in order to use at scale, organizations must integrate PGHD.^14^ Although researchers in each of these studies identified PGHD integration as a crucial need, none of them focused specifically on the integration of PGHD into the EHR. A focused review describing the integration of PGHD into the clinical environment is needed to map the successes and challenges of EHR integration. In turn, this may aid those developing future mHealth applications, designing EHR workflow integration, or implementing programs that will collect, use, and access PGHD.

We aimed to explore evidence to determine the extent of and to describe the nature of current PGHD integration into EHRs. The research question used to guide the inquiry was: What evidence has been reported on the integration of patient-generated health data into electronic health records?

## METHODS

We used methods based on the framework for scoping reviews described by Arksey and O’Malley and guidance from the Johanna Briggs Institute.^17^^,^^18^ Systematic scoping reviews can answer broad questions when evidence is deficient and may identify gaps in the research knowledge base.^19^ We published the study protocol before conducting this research.^20^

### Eligibility criteria

We included original articles that reported PGHD integration into EHRs for use by healthcare providers. We based the concept of PGHD on definitions from the ONC,^4^ but intentionally interpreted it to cover all types of health data including structured patient-reported outcomes (PROs). We defined EHR integration as automatic access to and availability of clinically relevant PGHD from the EHR without having to sign into a separate application (app). We included complete, partial, or in-progress integrations, but we excluded integrations described as a vision or “potential” integration. We also excluded chart reviews, opinion papers, case reports, and editorials. Conference proceedings, unpublished studies, and grey literature were eligible for inclusion. We included articles that reported PGHD integration to a patient portal if the study indicated that the data were visible within the EHR.

### Literature search

An information specialist (M.M.M.) developed the strategy for our primary database, MEDLINE, which entailed the review team pilot-testing a subset of results to ensure sensitivity, then translating the search for other databases. Information science colleagues conducted a peer review using PRESS guidelines of search strategies.^21^ The databases searched included Medline (Ovid) 1946–2019, Embase (embase.com) 1974–2019, CINAHL Complete (EBSCOhost) 1937–2019, Scopus (scopus.org) 1970–2019, Web of Science Core Collection (Clarivate Analytics) 1900–2019, Academic Search Ultimate (EBSCOhost) 1965–2019, Dissertations & Theses Global (ProQuest) 1861–2019, IEEE Xplore (IEEE.org) 1988–2019, and INSPEC (Elsevier.com) 1989–2019. No filters, such as date, language, or study type, were applied. The final search strategies are presented in Supplementary File S1. To search unpublished studies and grey literature, we used the Google search engine and limited the search to the first 50 results. Search terms included patient-generated health data, user-generated health data, self-tracking, integration, and electronic medical record.

### Study selection

Search results were imported into Covidence (Veritas Health Innovation, n.d.) systematic-review software for screening. Two trained nurse researchers (VLT and WH), conducted title, abstract, and full-text screening. Each researcher tested the screening criteria on a sample of titles and abstracts to ensure that the criteria were robust enough to capture eligible articles. If an abstract was not accompanied by the full text of the article, we contacted the authors by email. If we did not receive a response after 2 weeks, we reminded the authors a second time; if there was still no response, we excluded the abstract. In the second level of screening, the 2 researchers independently assessed the full text to determine eligibility. We recorded all reasons for full-text exclusion in Covidence and held regular meetings to achieve consensus.

### Data abstraction and analysis

We charted relevant data from the articles to predetermined extraction fields in a spreadsheet document and calculated frequencies for each field. We performed qualitative data analyses to determine technical integration, workflow integration, implementation details, challenges, and facilitators. We imported the data into Dedoose Version 7.0.23 (SocioCultural Research Consultants, n.d.), a qualitative data-analysis web app, and then configured the coding scheme, based on the extraction fields, into the application.^20^ To assess inter-rater reliability, 2 members of the research team (VLT and WH) independently categorized 10% of the articles against the coding scheme. Cohen’s kappa statistic value was 0.87, indicating good agreement.^22^ We proceeded with categorizing the entire set of articles and met weekly to reach consensus on any differences. We did not need assistance with unresolved disagreements.

After coding, we performed a conceptual thematic analysis.^23^ We created higher-level codes, categories, and themes related to the findings and used frequency analyses to describe the data. Finally, we identified themes and gaps in the literature, and implications for future work in this area.

## RESULTS

After removing duplicates in the search results, we screened 9463 titles and abstracts. The corpus included 2878 from MEDLINE (Ovid), 2118 from Embase, 2416 from CINAHL Complete (EBSCOhost), 1128 from Scopus, 135 from Web of Science Core Collection (Clarivate Analytics), 650 from Academic Search Ultimate (EBSCOhost), 120 from Dissertations & Theses Global (ProQuest), 17 from IEEE Xplore, and 18 from INSPEC. Of the 199 articles that underwent full-text screening, we included 17 articles. We added 2 additional articles to this review using the Google search for grey literature. The result was a total of 19 articles for the analysis.

**Figure 1.**
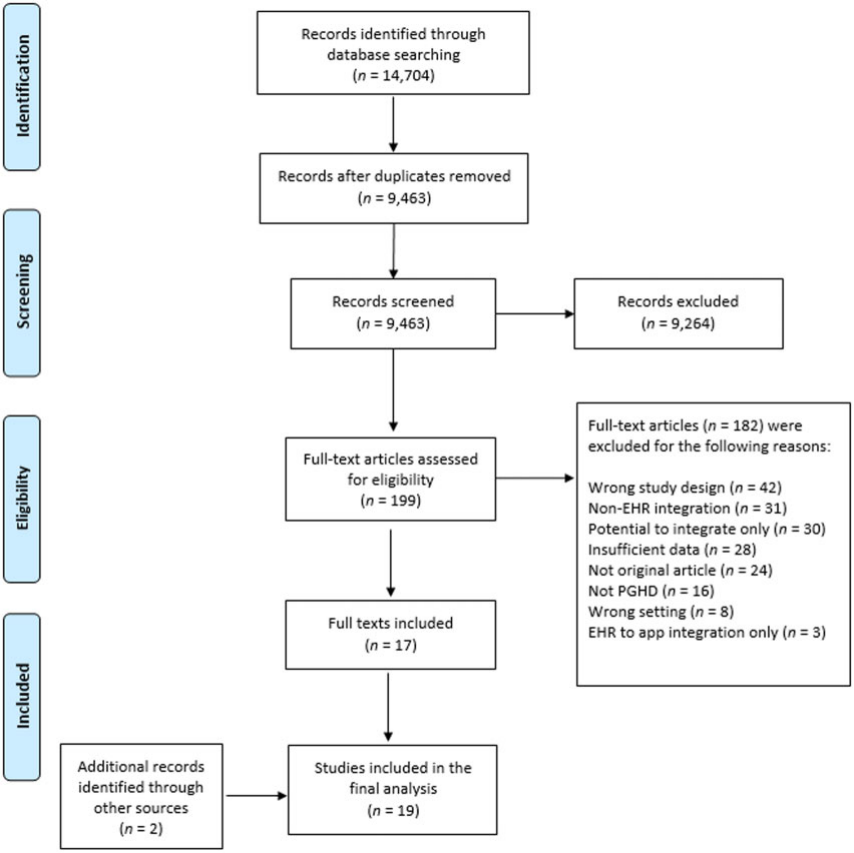
Flow diagram for study-selection process. *Note:* app, application; EHR, electronic health record; PGHD, patient-generated health data.

Most of the articles excluded in the title and abstract screening stage did not meet the definition of PGHD integration to EHRs. In the full-text screening stage, common reasons for exclusion were descriptions of prototypes that had not yet been implemented; opinions or editorials; and integration to a patient portal without mention of ability to review PGHD within the EHR. We excluded several articles because PGHD flowed from the EHR to a patient application instead of PGHD flowing into the EHR. Although examples of sharing data with patients were encouraging, this capability did not meet inclusion criteria unless the authors indicated bidirectional exchange. Figure 1 depicts the study-selection process, the full list of included articles is in Table 1, and the list of excluded articles is in Supplementary File S2.

**Table 1.** Included studies

Year of publication	Author(s)
2019	Absolom, Gibson, and Velikova
	Ancker, Mauer, Kalish, Vest, and Gossey
	Day et al
	Fisher et al
	Girgis, Durcinoska, Arnold, and Delaney
	Lewinski et al
	Zhang et al
2018	Gold et al
	Graetz et al
	Miyamoto, Dharmar, Fazio, Tang-Feldman, and Young Sharp
2017	Paterson, McAulay, and McKinstry
	Pennic
2016	Kumar, Goren, Stark, Wall, and Longhurst
	Sorondo et al
2015	Wagner et al
	Leventhal
2014	Moore et al
2013	Marquard et al

### Study characteristics

The year of publication ranged from 2013 to 2019 (Table 2). More than half of the studies were published after 2018, indicating that PGHD integration is a relatively new concept in research. We found a range of study evaluation designs and almost half of the studies consisted of pilots to examine feasibility or usability.^24^ The most prevalent study setting was outpatient, in a clinic or physician’s office (73.7%), followed by cancer centers (15.8%) and academic medical centers (10.5%), both considered inpatient settings. Many of the studies included patients who collected PGHD for one health condition, most commonly diabetes (42.1%). Further data on the study characteristics are in Supplementary File S3.

**Table 2.** Summary of study characteristics (*n *=* *19)

Characteristic	Number (%)
Year of publication	
2013	1 (5.3%)
2014	2 (10.5%)
2015	1 (5.3%)
2016	2 (10.5%)
2017	2 (10.5%)
2018	5 (26.3%)
2019	6 (31.6%)
Geographic region	
North America	16 (84.2%)
United Kingdom	2 (10.5%)
Australia	1 (5.3%)
Evaluation design	
Observational	3 (15.8%)
Descriptive	1 (5.3%)
Experimental	3 (15.8%)
Qualitative	3 (15.8%)
Mixed methods	3 (15.8%)
System description	6 (31.6%)
Study setting	
Outpatient or clinic	14 (73.7%)
Cancer center	3 (15.8%)
Academic medical center	2 (10.5%)
Target population	
Diabetes	8 (42.1%)
Cancer	4 (21.1%)
Hypertension	2 (10.5%)
Orthopedic surgery	2 (10.5%)
Multiple conditions	2 (10.5%)
Prostate-specific antigen screening	1 (5.3%)

### Integration characteristics

Across the 19 articles, 3 main types of PGHD as defined by Adler-Milstein and Nong^14^ were represented (Table 3). Over half of the studies (57.9%) collected patient activity or biometric data such as blood pressure or blood glucose. Several of the studies (36.8%) used PGHD in the form of questionnaire and survey responses, and one integrated PGHD related to health history. The prominent EHR for integration was Epic (63.2%); one study identified GE Centricity (5.3%), another identified MOSAIQ (5.3%), and all others did not specify the EHR (26.3%). Authors generally characterized the mode of PGHD transfer as active, indicating that the patient actively entered data, or passive, meaning that the device automatically downloaded the data without patient effort. More than half of the integrations (57.9%) reported active PGHD transfer, whereas 6 studies (31.6%) reported passive transfer and 2 (10.5%) reported both.

**Table 3.** Summary of integration characteristics

Characteristic	Number (%)
PGHD type	
Biometric and patient activity	11 (57.9%)
Questionnaires and surveys	7 (36.8%)
Health history	1 (5.3%)
EHR	
Epic	12 (63.2%)
Unidentified	5 (26.3%)
MOSAIQ	1 (5.3%)
GE centricity	1 (5.3%)
PGHD transfer	
Active	11 (57.9%)
Passive	6 (31.6%)
Both	2 (10.5%)
Developer platform	
Apple HealthKit	5 (26.3%)
Northwestern Medicine Patient-Reported Outcomes	2 (10.5%)
Microsoft HealthVault	1 (5.3%)
Validic	1 (5.3%)
Not reported	10 (52.6%)
Technical approach	
HL7	4 (21.1%)
APIs	3 (15.8%)
Bluetooth	2 (10.5%)
Not reported	10 (52.6%)

*Note:* APIs, application programming interfaces; HL7, Health Level 7; PGHD, patient-generated health data.

In addition to the EHR, some of the studies used a middleware platform to aggregate and assimilate PGHD. The most common platform used to share PGHD was Apple HealthKit (26.3%). Four studies (21.1%) reported the use of Health Level 7 (HL7) standards to transfer the PGHD; only one of those studies indicated the use of the Fast Healthcare Interoperability Resources (FHIR) specification, a recent HL7 standards framework created specifically to support mobile apps.^25^^,^^26^ Additional details on the integration characteristics are provided in Supplementary File S4.

### Thematic analysis

**Figure 2.**
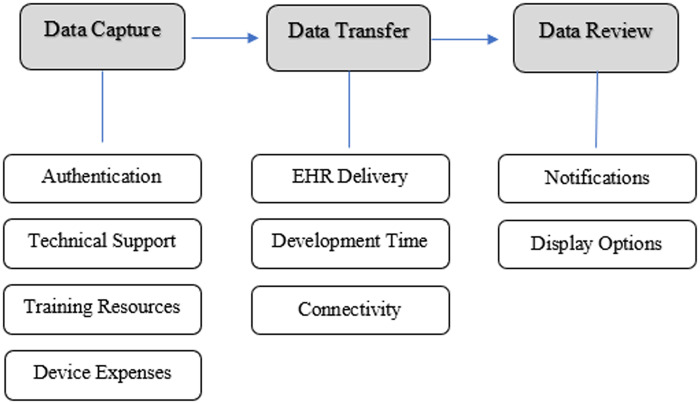
Themes related to patient-generated health data flow. *Note:* EHR, electronic health record.

We categorized themes (Figure 2) according to the steps of PGHD flow as described by Shapiro et al^27^ Data capture refers to methods to collect data and can involve multiple steps. Data transfer concerns communication of PGHD from the patient to the healthcare team. Data review refers to a member of the healthcare team deciding what to do with the data. For data capture and review, we specifically looked for themes that had a direct impact on integration. We describe the theme categorization and article attribution below and in Table 4.

**Table 4.** Thematic categorization and article attribution

Category	Theme	Number of articles
Data capture	Authentication	19
	Technical support	5
	Training resources	5
	Device expenses	4
Data transfer	EHR delivery	6
	Development time	6
	Connectivity	5
Data review	Notifications	7
	Display options	10

#### Data capture

Given the need for correct patient matching, the authentication and authorization process was a recurrent theme in every article. The most common method described was a patient portal request to the patient to authorize data transfer or capture consent. Other methods included email, text messaging, or the mHealth app itself to prompt the patient for PGHD. One article was less specific, but indicated use of multiple authentication protocols to authorize collection and integration.^28^ Leventhal^25^ described portal use in a positive light, explaining that the design puts patients in control of their data. However, many of the articles raised the concern for patients without a portal account or previous contact with the provider or hospital. As a potential solution, one article described a high-touch approach that provides full onboarding support including delivery of a device to the home once PGHD use is ordered.^29^

Across the article corpus, authors emphasized resource requirements for in 3 areas: personnel for technical support, training resources, and device expenses. Types of resources related to the patient consisted of a telephone support line, development of support materials, and home visits. One implementation utilized nurse case managers for problem solving with patients.^30^ Another identified technical support as a barrier to provider adoption, indicating that providers were answering emails from patients related to troubleshooting and technical support issues rather than focusing on clinical questions.^31^

Training was a recurrent theme related to integration success, especially training for patients on how to use devices, format text messages, and access patient portals. One article indicated that the face-to-face training sessions were a fundamental part of the intervention’s success.^32^ Other studies found onboarding sessions, patient instruction manuals, and other device-support materials given to patients before participating in PGHD integration activities to be critical. Fisher et al^33^ described a program with sustainable scalability that used nonlicensed patient navigators to provide patient-facing education.

Several of the articles reported that PGHD devices were expensive for both acquisition and management. In some cases, there was concern over the additional expense of Apple HealthKit-enabled devices, especially for patients without means. Overall, the authors expressed optimism for changes to national reimbursement models that may include PGHD as a billable service or that encourage payer subsidization of device costs.

#### Data transfer

EHR delivery of PGHD used a variety of platforms, standards, and methods. Other reviews have reported the theme of facilitating the passive transfer of PGHD; but referred to it in the context of the patient burden of uploading or inputting data.^15^^,^^16^^,^^34^ Authors of several of the articles in this review described the transfer burden for the provider or organization.^35–40^ Provider work was required to file data to the correct patient, actively link to the patient record, or match to the patient ID. One article described the limitation of being able to link the patient data to only a single provider.^31^ In some cases, PGHD were automatically uploaded or automatically inserted into the EHR. Kumar et al^35^ concluded that the passive delivery of PGHD to the EHR facilitates more efficient provider workflows.

Development time was another recurrent theme regarding PGHD integration. One article reported that developing the interface between the portal and the EHR took 6 months and in another, the development work took 4 years.^31^^,^^32^ One article noted a 3.5-year timeline due to the creation of a secure architecture compatible with the EHR vendor-proprietary non-FHIR web services.^41^ Setup time with patients added 45–60 min to a scheduled visit according to another article, and in others, time was needed to develop customized flowsheets, create patient registries, and identify relevant evidence for the use of PGHD in clinical care.^33^^,^^35^^,^^36^^,^^42^

Multiple articles reported connectivity issues that impacted the integration to the EHR along with other issues such as updates to software, the EHR, APIs, and PRO instruments.^25^^,^^30^^,^^43^ In addition, several articles identified compatibility limitations between devices and specific Internet browsers.^31^^,^^37^ In some cases, the portal behaved differently with some operating system and browser combinations. Transmission over commercial networks and home connectivity for patients was problematic for some PGHD integrations.

#### Data review

EHR notifications and display options emerged as themes relevant to provider review of PGHD. In several studies, alerts were sent by email or text message for concerning symptoms or patient questions.^32^^,^^39^^,^^44^^,^^45^ In return, providers could message patients. Organizations could also tailor alerts and messages to provider preferences. Some integrations used dashboards to highlight patients who required attention, and Sorondo et al^31^ described use of a coordinator to triage results before sending them to the provider. Providers also received notifications for compliance purposes.

Dashboards and structured reports were frequently described in the articles as display options for the provider view of PGHD during the visit. The most common data views were a tabular or graphic format, and visualization of data trends where possible. Several of the Epic integrations used the Epic Synopsis report, a graphical display. However, some authors noted that providers did not overtly refer to PGHD, seldom used PGHD in everyday care, and did not embrace routine PRO use citing to a lack of actionable data.^40^ Many integration examples saved the PGHD to flowsheets only and did not permit the inclusion of PGHD into provider notes. One article discussed strategies to incentivize providers with the provision of actionable recommendations to respond to PGHD.

## DISCUSSION

Currently, PGHD appear to be seldom incorporated into clinical care, resulting in a missed opportunity to close information gaps. In this scoping review, we found few studies providing details on the integration of PGHD into EHRs. These results suggest that PGHD integrations are in their infancy or are underreported in the literature. Almost half of the studies were in the pilot phase and few measured outcomes, suggesting that development and testing are still at a preliminary stage. Best practices on how to incorporate PGHD into clinical workflows are not yet available.

There was little representation from inpatient care settings in this review, which may indicate that longitudinal care initiatives are mostly making use of PGHD. More than half of the articles reported integration with Epic EHR, which is not surprising given that Epic has the largest share of the ambulatory EHR market.^46^ Given the number of mHealth apps that collect PGHD, there are relatively few EHR vendors with reported integrations.

However, the growth of PGHD literature in recent years is promising. Of the 19 studies included, 11 were published since January 2018. This growth may be due to increased availability of standards-based APIs such as FHIR and new legislation to discourage information blocking.^7^ Another factor may be the growing movement toward consumer access to their health data spurred by organizations such as the CARIN Alliance and projects such as GetMyHealthData seeking to redesign digital health-data sharing processes.^3^ In the coming years, we expect to see more PGHD integration to EHRs and digitally sharing EHR data with patients.

### Impact of data capture on PGHD integration

Patients with a portal account for their provider or hospital may be tied to particular EHRs, devices or apps. This is somewhat limiting and may be difficult for a patient to know which device to choose. It may also involve significant training-resource requirements due to the patient’s lack of understanding when it comes to health data and technology.^32^^,^^33^^,^^37^ To fully understand PGHD integration, our findings suggest that it is essential to explore PGHD integration in the context of a variety of data sources and EHRs. We believe that advances in passive data collection may reduce the expertise required to capture PGHD.

Blood pressure and continuous blood-glucose monitoring devices were described as expensive for the patients who may need the technology the most. Given the growing trend in disparities, with critical-access hospitals as noted laggards, PGHD efforts that widen the digital divide are of great concern.^47–49^ Our findings indicate that the evaluation of PGHD interventions would benefit from a closer examination of financial implications for patients, reimbursement models, and the impact on health equity.

### Impact of data transfer on PGHD integration

Overall, most of the articles provided little technical content, making it difficult to find commonalities or identify best practices. Despite the lack of descriptive content, this review exposed the operational challenges of integrating PGHD. Specifically, we found that additional work to match PGHD to patient charts, create new interfaces, or hire extra staff all contributed to the success of the integrations—or lack thereof. Wider adoption of new interoperability standards and standardized APIs to simplify integration may help to decrease the EHR delivery burden and enhance the long-term sustainability of PGHD initiatives. To our knowledge, the EHR delivery component of PGHD integration is not routinely acknowledged and warrants further attention.

### Impact of data review on PGHD integration

Research suggests that the provider documentation burden is growing.^12^ To make data actionable within clinical workflows, PGHD data not only need to be present in the EHR but also in the locations where the data will be seen and used. Adding PGHD to the EHR requires careful consideration, and the data must be accessible to all members of the healthcare team. From this review, it remains unclear if this requires limits on which PGHD to integrate, if there should be a review process before inclusion, or if clinicians require certain levels of summarization of the PGHD data. PGHD data review could also benefit from standardized practices around data visualization, filters, and notifications.

### Recommendations for implementers and researchers

Given the examples of PGHD integration found, we provide the following recommendations for implementers and propose considerations for future research. First, we recommend that researchers outline the detailed technical aspects of PGHD implementation in publications, including the specification of interfaces. Sharing such details may provide a better understanding of what is working optimally. Second, the time and resource commitment to design, develop, and implement PGHD integration should undergo careful consideration. We recommend adoption of standard data interfaces, ideally compliant with the substitutable medical applications and reusable technologies (SMART) specification for transparent authentication and the FHIR specification for data exchange.^26^ When possible, methods should be employed to decrease effort by the provider or organization for data transfer. Third, similar to the lessons learned from EHR implementations, technical analysts should conduct a thorough examination of provider workflows in order to assimilate PGHD for clinical decisions. Time is required to understand clinician visualization preferences, and how to summarize and visually display PGHD alongside other EHR data to facilitate actionable use by providers.

We propose multiple areas for further exploration. In order to compare integration methods, research to examine varied types of PGHD, with various platforms, would be beneficial. Evidence is needed to answer questions regarding which platforms work best, whether the FHIR standards are ready, what PGHD tools are ready, and what additional development work is needed.^50^

The generation of evidence on sustainable payment models is needed to subsidize device costs for patients and to reimburse providers for PGHD review. In addition, methods to incentivize providers and patients are worth exploration. Although excluded from this review, it would also be worthwhile to examine the flow of clinical data from EHRs to the patient, to understand if this data flow creates similar integration issues or if it facilitates shared decision-making when patients have all of their data in one place.

### Limitations

Although we employed a rigorous methodological approach, this review has limitations. Despite the comprehensive search across multiple platforms, we likely did not capture all articles related to PGHD. Notably, the Medical Subject Heading (MeSH) term in PubMed for PGHD was created only in January 2018, making the search for scientific works before then more challenging. We attempted to mitigate the small sample size with the inclusion of grey literature (Google), assuming that the most recent examples may not yet be published in journals. As part of the scoping-review framework, Arksey and O’Malley^17^ suggested consultation with stakeholders to inform study findings. Stakeholder consultation for this review was limited to informatics experts on the study team. Lastly, we did not conduct a formal assessment for article quality or risk of reporting bias because we intended to report all evidence, regardless of quality.^51^

## CONCLUSION

PGHD are potentially valuable contributions to the patient story that can help to close healthcare gaps and support personalized medicine. This review underscores the need for best practices and better reporting of technical requirements to integrate PGHD into EHRs for the optimization of clinical care, in order to leverage the potential value of PGHD.

## FUNDING

Dr. Victoria L. Tiase is supported by the Jonas Nurse Leaders Scholar Program (Jonas Philanthropies). Content is solely the responsibility of the authors and does not necessarily represent the official views of Jonas Philanthropies. This work was supported in part by a Western Institute of Nursing/Council for the Advancement of Nursing Science Dissertation Award.

## AUTHOR CONTRIBUTIONS

VLT designed the review, developed the research question, and contributed meaningfully to the drafting and editing. MMM conducted the literature search. VLT and WH performed both levels of screening. WH conducted the thematic analysis with VLT and contributed meaningfully to the drafting and editing. KAS, GDF, CS, CW, and MRC served as informatics domain stakeholders and critically revised the article. All authors gave final approval of the version to be published.

## SUPPLEMENTARY MATERIAL

Supplementary material is available at *Journal of the American Medical Informatics Association* online.

## CONFLICT OF INTEREST STATEMENT

None declared.

## Supplementary Material

ooaa052_Supplementary_Data
