# Supplementary material for: Patient-generated health data and electronic health record integration: a scoping review
Source: JAMIA Open. 2020 Dec 5;3(4):619–27. doi: 10.1093/jamiaopen/ooaa052 (PMC7969964; doi:10.1093/jamiaopen/ooaa052)
Supplement: ooaa052_Supplementary_Data [file ooaa052_supplementary_data.zip › Supplementary File 1.docx]

Supplementary File 1

Search Strategies by Database

**Ovid MEDLINE(R) August 2, 2019**

1 Patient Generated Health Data/ (38)

2 ("patient generated data" or "patient generated health data" or "patient-generated data" or "patient-generated health data" or (("patient generated" or "patient-generated") adj3 (data? or dataset? or "data set?"))).ti,ab,kf,kw. (188)

3 ((patient* or caregiver*) adj2 (generated or recorded) adj3 (data? or dataset? or "data set?")).ti,ab,kf,kw. (1007)

4 or/1-3 [PGHD set1] (1035)

5 (("user generated" or user-generated) adj3 (data? or "data set?" or data-set?)).ti,ab,kf,kw. (45)

6 ("self recorded health data" or "self-recorded health data" or (("self-recorded" or "self recorded" or "self tracking" or self-tracking or "self tracke??" or self-tracke?? or "self-expressed" or "self expressed" or "personally collected") adj3 (data? or dataset? or "data set?"))).ti,ab,kf,kw. (58)

7 ((personal* or self or patient*) adj2 data* adj2 (tracking or tracke??)).ti,ab,kf,kw. (82)

8 ("quantified self" or lifelog).ti,ab,kf,kw. (122)

9 or/5-8 [PGHD set 2] (280)

10 Telemedicine/ (19941)

11 (mHealth or m-health or "mobile health" or ehealth or e-health or "smart technolog*" or smartphone* or "smart phone*" or smartwatch* or "smart watch*").ti,ab,kf,kw. (19156)

12 or/10-11 [mhealth. PGHD set 3] (35286)

13 Self Report/ or ("self report*" or self-report*).ti,ab,kf,kw. (152026)

14 self care/ or self-management/ (32935)

15 ("self care" or "self management" or "self monitor*" or self-monitor*).ti,ab,kf,kw. (38860)

16 or/13-15 [ self care/management/monitor ] (205737)

17 16 and (technolog* or device* or wearable*).ti,ab,kf,kw. [ self care/mgt/monitor + tech Set ] (9011)

18 Wearable Electronic Devices/ (1217)

19 (((body-worn or "body worn" or wearable*) adj2 (biosensor* or sensor* or device? or technolog*)) or (electronic adj1 skin)).ti,ab,kf,kw. (5464)

20 or/18-19 [wearable devices set ] (6002)

21 Monitoring, Ambulatory/ (7778)

22 (monitoring adj1 (outpatient* or patient* or ambulatory)).ti,ab,kf,kw. (9088)

23 or/21-22 [ambulatory monitoring set ] (16479)

24 patient reported outcome measures/ (3549)

25 ("patient reported" adj4 outcome*).ti,ab,kf,kw. (16952)

26 or/24-25 [ patient reported outcome set ] (17809)

27 or/4,9,12,17,20,23,26 [ all PGHD sets combined ] (80242)

28 medical record linkage/ or medical records systems, computerized/ or health information exchange/ (23092)

29 ((computer* or electronic or linkage) adj2 (health or medical) adj2 record?).ti,ab,kf,kw. or ehr.ti,ab. (30532)

30 "Meaningful Use"/ or (meaningful adj1 "use?").ti,ab,kf,kw. (1824)

31 or/28-30 [EHR set 1] (50039)

32 medical records/ or medical records, problem-oriented/ (65892)

33 information systems/ or big data/ or community networks/ or geographic information systems/ or health information systems/ or knowledge bases/ or biological ontologies/ or gene ontology/ or medical informatics computing/ or public health informatics/ (42946)

34 database management systems/ or data systems/ (7635)

35 Automation/ (17263)

36 32 and (or/33-35) [ automat* medical records - EMR set ] (1758)

37 or/31,36 [EHR or EMR set] (51622)

38 *Decision Support Systems, Clinical/ or (CDS or CDSS).ti,ab. or ((clinical or hospital) adj4 ("decision support system?" or "information system?")).ti,ab. (23093)

39 (("patient* portal*" or patient*) adj2 portal*).ti,ab,kf,kw. (4401)

40 and/27,37 [ PGHD + EHR/EMR set] (2342)

41 (and/27,38) not 40 [PGHD + CDSS set] (424)

42 (and/27,39) not (40 or 41) [PGHD + Patient Portal set] (118)

43 or/40-42 [ Final Set] (2884)

44 remove duplicates from 43 (2878)

**Embase (embase.com) August 9, 2019**

#43 #40 OR #41 OR #42 3593

#42 #28 AND #39 NOT (#40 OR #41) 175

#41 #28 AND #38 NOT #40 299

#40 #28 AND #35 3119

#39 (('patient* portal*' OR patient*) NEAR/2 portal*):ti,ab,kw 6551

#38 #36 OR #37 20466

#37 'clinical decision support system'/de OR cds:ti,ab OR cdss:ti,ab OR (((clinical OR hospital) NEAR/4 ('decision support system?' OR 'information system?')):ti,ab,kw) 20466

#36 'clinical decision support system'/de 2365

#35 #31 OR #34 78122

#34 #32 AND #33 7853

#33 'information system'/de OR 'decision support system'/exp/mj OR 'hospital information system'/exp/mj OR 'medical information system'/mj OR 'nursing information system'/mj OR 'automation'/mj 80653

#32 'medical record'/de 171140

#31 #29 OR #30 236339

#30 ((computer* OR electronic OR linkage) NEAR/2 (health OR medical) NEAR/2 record?):ti,ab,kw 28527

#29 'electronic health record'/de OR 'meaningful use criteria'/de OR 'electronic medical record'/de OR 'electronic patient record'/de 63978

#28 #3 OR #8 OR #11 OR #16 OR #19 OR #23 OR #26 105463

#27 #3 OR #8 OR #11 OR #16 OR #19 OR #26 89781

#26 #24 OR #25 33477

#25 ('patient reported' NEAR/4 outcome*):ti,ab,kw 29160

#24 'patient-reported outcome'/de 16356

#23 #20 OR #21 OR #22 17182

#22 (monitoring NEAR/1 (outpatient* OR patient* OR ambulatory)):ti,ab,kw 13838

#21 (monitoring NEAR/1 (outpatient* OR patient* OR ambulatory)):ti,ab,kw 13838

#20 'ambulatory monitoring'/mj 3930

#19 #16 OR #17 OR #18 18663

#18 ((('body worn' OR 'body worn' OR wearable*) NEAR/2 (biosensor* OR biometric* OR sensor* OR device? OR technolog*)):ti,ab,kw) OR ((electronic NEAR/3 skin):ti,ab,kw) 5311

#17 'electronic device'/exp AND ('body worn*':ti,ab,kw OR 'body worn':ti,ab,kw OR wearable*:ti,ab,kw) 2485

#16 #15 AND (technolog*:ti,ab,kw OR device*:ti,ab,kw OR wearable*:ti,ab,kw) 12539

#15 #12 OR #13 OR #14 286030

#14 'self care':ti,ab,kw OR 'self management':ti,ab,kw OR 'self monitor*':ti,ab,kw 55772

#13 'self care'/de 53739

#12 'self report'/de OR 'self report*':ti,ab,kw 214338

#11 #9 OR #10 40921

#10 mhealth:ti,ab,kw OR 'm health':ti,ab,kw OR 'mobile health':ti,ab,kw OR ehealth:ti,ab,kw OR 'e health':ti,ab,kw OR 'smart technolog*':ti,ab,kw OR smartphone*:ti,ab,kw OR 'smart phone*':ti,ab,kw OR smartwatch*:ti,ab,kw OR 'smart watch*':ti,ab,kw 24142

#9 'telemedicine'/exp/mj 18599

#8 #5 OR #6 OR #7 211

#7 'quantified self':ti,ab,kw OR lifelog:ti,ab,kw 120

#6 ((personal* OR self OR patient*) NEAR/2 data* NEAR/2 (tracking OR tracke??)):ti,ab,kw 92

#5 'self recorded health data':ti,ab,kw OR 'self-recorded health data':ti,ab,kw OR ((('self-recorded' OR 'self recorded' OR 'self tracking' OR 'self tracking' OR 'self tracke??' OR 'self tracke??' OR 'self-expressed' OR 'self expressed' OR 'personally collected') NEAR/3 (data? OR dataset? OR 'data set?')):ti,ab,kw) 2

#4 (('user generated' OR 'user generated') NEAR/3 (data? OR 'data set?' OR 'data set?')):ti,ab,kw 6

#3 #1 OR #2 188

#2 ((patient* OR caregiver*) NEAR/2 (generated OR recorded) NEAR/3 (data? OR dataset? OR 'data set?')):ti,ab,kw 19

#1 'patient generated data':ti,ab,kw OR 'patient generated health data':ti,ab,kw OR 'patient-generated data':ti,ab,kw OR 'patient-generated health data':ti,ab,kw OR ((('patient generated' OR 'patient-generated') NEAR/3 (data? OR dataset? OR 'data set?')):ti,ab,kw) 169

**CINAHL Complete (Ebscohost) August 19, 2019**

S34 S29 OR S30 OR S31 OR S32 OR S33 3,288

S33 S19 AND (S28) 118

S32 S19 AND (S27) 439

S31 S19 AND (S26) 57

S30 S19 AND (S22) 58

S29 S19 AND (S20 or S21) 2,894

S28 TI ( (("patient* portal*" or patient*) N2 portal*) ) OR AB ( (("patient* portal*" or patient*) N2 portal*) ) 1,114

S27 (MH "Decision Support Systems, Clinical") OR TI ( (CDS or CDSS) ) OR AB ( (CDS or CDSS) ) OR TI ( ((clinical or hospital) N4 ("decision support system?" or "information system?")) ) OR AB ( ((clinical or hospital) N4 ("decision support system?" or "information system?")) ) 6,443

S26 S23 and (S24 or S25) 1,217

S25 (MH "Management Information Systems") OR (MH "Automation") 6,930

S24 (MH "Information Systems") OR (MH "Health Information Systems+") OR (MH "Management Information Systems") 54,457

S23 (MH "Medical Records") OR (MH "Problem Oriented Records") 18,351

S22 (MH "Meaningful Use") OR TI (meaningful N1 "use?") OR AB (meaningful N1 "use?") 1,619

S21 TI ( ((computer* or electronic or linkage) N2 (health or medical) N2 record?) ) OR AB ( ((computer* or electronic or linkage) N2 (health or medical) N2 record?) ) 14,551

S20 (MH "Electronic Health Records") OR (MH "Medical Record Linkage") OR (MH "Health Information Systems") OR (MH "Clinical Information Systems") OR (MH "Patient Record Systems") OR (MH "Patient Portals") OR (MH "Electronic Data Interchange") OR (MH "Health Level 7") OR (MH "National Health Information Network") 38,831

S19 S3 OR S7 OR S10 OR S16 OR S17 OR S18 63,951

S18 (MH "Patient-Reported Outcomes") OR TI ("patient reported" N4 outcome*) OR AB ("patient reported" N4 outcome*) 8,857

S17 TI ( (((body-worn or "body worn" or wearable*) N2 (biosensor* or sensor* or device? or technolog*)) or (electronic N1 skin)) ) OR AB ( (((body-worn or "body worn" or wearable*) N2 (biosensor* or sensor* or device? or technolog*)) or (electronic N1 skin)) ) OR TX ( (((body-worn or "body worn" or wearable*) N2 (biosensor* or sensor* or device? or technolog*)) or (electronic N1 skin)) ) 3,856

S16 S14 AND S15 5,289

S15 TI ( (technolog* or device* or wearable*) ) OR AB ( (technolog* or device* or wearable*) ) 170,718

S14 (S11 OR S12 OR S13) 155,191

S13 ("self care" or "self management" or "self monitor*" or self-monitor*) 52,763

S12 (MH "Self Care") OR (MH "Self-Management") 34,811

S11 (MH "Self Report") OR TI ( ("self report*" or self-report*) ) OR AB ( ("self report*" or self-report*) ) 105,081

S10 S8 OR S9 17,592

S9 TI ( (mHealth or m-health or "mobile health" or ehealth or e-health or "smart technolog*" or smartphone* or "smart phone*" or smartwatch* or "smart watch*") ) OR AB ( (mHealth or m-health or "mobile health" or ehealth or e-health or "smart technolog*" or smartphone* or "smart phone*" or smartwatch* or "smart watch*") ) 9,339

S8 (MH "Telemedicine") 9,398

S7 S4 OR S5 OR S6 104

S6 TI ( ("quantified self" or lifelog) ) OR AB ( ("quantified self" or lifelog) ) 42

S5 TI ( ((personal* or self or patient*) N2 data* N2 (tracking or tracke??)) ) OR AB ( ((personal* or self or patient*) N2 data* N2 (tracking or tracke??)) ) 46

S4 TI ( ("self recorded health data" or "self-recorded health data" or (("self-recorded" or "self recorded" or "self tracking" or self-tracking or "self tracke??" or self-tracke?? or "self-expressed" or "self expressed" or "personally collected") N3 (data? or dataset? or "data set?"))) ) OR AB ( ("self recorded health data" or "self-recorded health data" or (("self-recorded" or "self recorded" or "self tracking" or self-tracking or "self tracke??" or self-tracke?? or "self-expressed" or "self expressed" or "personally collected") N3 (data? or dataset? or "data set?"))) ) 25

S3 S1 OR S2 30,360

S2 ((patient* or caregiver*) N2 (generated or recorded) N3 (data? or dataset? or "data set?")) 30,173

S1 TI ( ("patient generated data" or "patient generated health data" or "patient-generated data" or "patient-generated health data" or (("patient generated" or "patient-generated") N3 (data? or dataset? or "data set?"))) ) OR AB ( ("patient generated data" or "patient generated health data" or "patient-generated data" or "patient-generated health data" or (("patient generated" or "patient-generated") N3 (data? or dataset? or "data set?"))) ) OR TX (("patient generated data" or "patient generated health data" or "patient-generated data" or "patient-generated health data" or (("patient generated" or "patient-generated") N3 (data? or dataset? or "data set?"))) ) 266

**Scopus (scopus.com) August 19, 2019**

( ( TITLE-ABS-KEY ( ( "patient generated data" OR "patient generated health data" OR "patient-generated data" OR "patient-generated health data" OR ( ( "patient generated" OR "patient-generated" ) W/3 ( data? OR dataset? OR "data set?" ) ) ) ) ) OR ( TITLE-ABS-KEY ( ( ( "user generated" OR user-generated ) W/3 ( data? OR "data set?" OR data-set? ) ) ) ) OR ( TITLE-ABS-KEY ( ( "self recorded health data" OR "self-recorded health data" OR ( ( "self-recorded" OR "self recorded" OR "self tracking" OR self-tracking OR "self tracke??" OR self-tracke?? OR "self-expressed" OR "self expressed" OR "personally collected" ) W/3 ( data? OR dataset? OR "data set?" ) ) ) ) ) OR ( TITLE-ABS-KEY ( ( "quantified self" OR lifelog ) ) ) OR ( TITLE-ABS-KEY ( mhealth OR m-health OR "mobile health" OR ehealth OR e-health OR "smart technolog*" OR smartphone* OR "smart phone*" OR smartwatch* OR "smart watch*" ) ) OR ( TITLE-ABS-KEY ( mhealth OR m-health OR "mobile health" OR ehealth OR e-health OR "smart technolog*" OR smartphone* OR "smart phone*" OR smartwatch* OR "smart watch*" ) ) OR ( TITLE-ABS-KEY ( "self report*" OR self-report* OR "self care" OR "self management" OR "self monitor*" OR self-monitor* ) AND TITLE-ABS-KEY ( technolog* OR device* OR wearable* ) ) OR ( TITLE-ABS-KEY ( ( ( ( body-worn OR "body worn" OR wearable* ) W/2 ( biosensor* OR sensor* OR device? OR technolog* ) ) OR ( electronic W/1 skin ) ) ) ) OR ( TITLE-ABS-KEY ( "patient reported" W/4 outcome* ) OR TITLE-ABS-KEY ( ( ( ( body-worn OR "body worn" OR wearable* ) W/2 ( biosensor* OR sensor* OR device? OR technolog* ) ) OR ( electronic W/1 skin ) ) ) ) ) AND ( TITLE-ABS-KEY ( ( ( computer* OR electronic OR linkage ) W/2 ( health OR medical ) W/2 record? ) ) OR TITLE-ABS ( ehr ) )

2,647 document results

***Web of Science Core Collection* (Clarivate Analytics) August 19, 2019**

# 18 761 #17 OR #15 OR #14 OR #13

# 17 182 #16 AND #8

# 16 4,997 TS= (("patient* portal*" or patient*) NEAR/2 portal*)

# 15 123 #12 AND #8

# 14 4 #10 AND #8

# 13 526 #9 AND #8

# 12 3,197 #10 OR #11

# 11 3,028 TOPIC: (((clinical or hospital) NEAR/4 ("decision support system?" or "information system?")))

# 10 169 TOPIC: ((meaningful NEAR/1 "use?"))

# 9 16,574 TS=(((computer* or electronic or linkage) NEAR/2 (health or medical) NEAR/2 record?))

# 8 49,164 #7 OR #6 OR #5 OR #4 OR #3 OR #2 OR #1

# 7 9,922 TOPIC: ((((body-worn or "body worn" or wearable*) NEAR/2 (biosensor* or sensor* or device? or technolog*)) or (electronic NEAR/1 skin)))

# 6 9,320 TOPIC: (("self report*" or self-report* or "self care" or "self management" or "self monitor*" or self-monitor*) and (technolog* or device* or wearable*))

# 5 32,138 TOPIC: ((mHealth or m-health or "mobile health" or ehealth or e-health or "smart technolog*" or smartphone* or "smart phone*" or smartwatch* or "smart watch*"))

# 4 194 TOPIC: (((personal* or self or patient*) NEAR/2 data* NEAR/2 (tracking or tracke??)))

# 3 7 TOPIC: (("self recorded health data" or "self-recorded health data" or (("self-recorded" or "self recorded" or "self tracking" or self-tracking or "self tracke??" or self-tracke?? or "self-expressed" or "self expressed" or "personally collected") NEAR/3 (data? or dataset? or "data set?"))))

# 2 61 TOPIC: (((patient* or caregiver*) NEAR/2 (generated or recorded) NEAR/3 (data? or dataset? or "data set?")))

# 1 124 TS=("patient generated data" or "patient generated health data" or "patient-generated data" or "patient-generated health data" or (("patient generated" or "patient-generated") NEAR/3 (data? or dataset? or "data set?")))

**Academic Search Ultimate (Ebscohost) August 19, 2019**

S25 S21 OR S22 OR S23 OR S24 1,403

S24 S14 AND S20 81

S23 S14 AND S19 81

S22 S14 AND S18 12

S21 S14 AND S17 1,279

S20 TI ( ( (("patient* portal*" or patient*) N2 portal*) ) ) OR AB ( ( (("patient* portal*" or patient*) N2 portal*) ) ) OR KW ( ( (("patient* portal*" or patient*) N2 portal*) ) ) 1,764

S19 TI ( ( ((clinical or hospital) N4 ("decision support system?" or "information system?")) ) OR AB ( ( ((clinical or hospital) N4 ("decision support system?" or "information system?")) ) OR KW ( ( ((clinical or hospital) N4 ("decision support system?" or "information system?")) ) 1,607

S18 TI (meaningful N1 "use?") OR AB (meaningful N1 "use?") OR KW (meaningful N1 "use?") 864

S17 S15 OR S16 20,394

S16 TI ( ( ((computer* or electronic or linkage) N2 (health or medical) N2 record?) ) ) OR AB ( ( ((computer* or electronic or linkage) N2 (health or medical) N2 record?) ) ) OR KW ( ( ((computer* or electronic or linkage) N2 (health or medical) N2 record?) ) ) 16,452

S15 DE "ELECTRONIC health records" 11,101

S14 S3 OR S4 OR S5 OR S6 OR S7 OR S8 OR S11 OR S12 OR S13 60,198

S13 TI ("patient reported" N4 outcome*) OR AB ("patient reported" N4 outcome*) OR KW ("patient reported" N4 outcome*) 9,713

S12 TI ( ( (((body-worn or "body worn" or wearable*) N2 (biosensor* or sensor* or device? or technolog*)) or (electronic N1 skin)) ) ) OR AB ( ( (((body-worn or "body worn" or wearable*) N2 (biosensor* or sensor* or device? or technolog*)) or (electronic N1 skin)) ) ) OR KW ( ( (((body-worn or "body worn" or wearable*) N2 (biosensor* or sensor* or device? or technolog*)) or (electronic N1 skin)) ) ) 5,655

S11 S9 AND S10 720

S10 TI ( (technolog* or device* or wearable*) ) OR AB ( (technolog* or device* or wearable*) ) OR KW ( (technolog* or device* or wearable*) ) 1,547,951

S9 (DE "HEALTH self-care") OR (DE "PATIENT self-monitoring") 11,478

S8 TI ( ( (mHealth or m-health or "mobile health" or ehealth or e-health or "smart technolog*" or smartphone* or "smart phone*" or smartwatch* or "smart watch*") ) ) OR AB ( ( (mHealth or m-health or "mobile health" or ehealth or e-health or "smart technolog*" or smartphone* or "smart phone*" or smartwatch* or "smart watch*") ) ) OR KW ( ( (mHealth or m-health or "mobile health" or ehealth or e-health or "smart technolog*" or smartphone* or "smart phone*" or smartwatch* or "smart watch*") ) ) 34,767

S7 DE "TELEMEDICINE" 10,945

S6 TI ( ( ("quantified self" or lifelog) ) ) OR AB ( ( ("quantified self" or lifelog) ) ) OR KW ( ( ("quantified self" or lifelog) ) ) 183

S5 TI ( ( ((personal* or self or patient*) N2 data* N2 (tracking or tracke??)) ) ) OR AB ( ( ((personal* or self or patient*) N2 data* N2 (tracking or tracke??)) ) ) OR KW ( ( ((personal* or self or patient*) N2 data* N2 (tracking or tracke??)) ) ) 77

S4 TI ( ( ("self recorded health data" or "self-recorded health data" or (("self-recorded" or "self recorded" or "self tracking" or self-tracking or "self tracke??" or self-tracke?? or "self-expressed" or "self expressed" or "personally collected") N3 (data? or dataset? or "data set?"))) ) ) OR AB ( ( ("self recorded health data" or "self-recorded health data" or (("self-recorded" or "self recorded" or "self tracking" or self-tracking or "self tracke??" or self-tracke?? or "self-expressed" or "self expressed" or "personally collected") N3 (data? or dataset? or "data set?"))) ) ) OR KW ( ( ("self recorded health data" or "self-recorded health data" or (("self-recorded" or "self recorded" or "self tracking" or self-tracking or "self tracke??" or self-tracke?? or "self-expressed" or "self expressed" or "personally collected") N3 (data? or dataset? or "data set?"))) ) ) 36

S3 (S1 OR S2) 932

S2 TI ( ((patient* or caregiver*) N2 (generated or recorded) N3 (data? or dataset? or "data set?")) ) OR AB ( ((patient* or caregiver*) N2 (generated or recorded) N3 (data? or dataset? or "data set?")) ) OR KW ( ((patient* or caregiver*) N2 (generated or recorded) N3 (data? or dataset? or "data set?")) ) 932

S1 TI ( ( ("patient generated data" or "patient generated health data" or "patient-generated data" or "patient-generated health data" or (("patient generated" or "patient-generated") N3 (data? or dataset? or "data set?"))) ) ) OR AB ( ( ("patient generated data" or "patient generated health data" or "patient-generated data" or "patient-generated health data" or (("patient generated" or "patient-generated") N3 (data? or dataset? or "data set?"))) ) ) OR KW ( ( ("patient generated data" or "patient generated health data" or "patient-generated data" or "patient-generated health data" or (("patient generated" or "patient-generated") N3 (data? or dataset? or "data set?"))) ) ) 51

**Dissertations & Theses Global (ProQuest) August 19, 2019**

S20 S16 or S17 or S18 or S19 123

S19 S11 and S15 10

S18 S11 and S14 23

S17 S11 and S13 7

S16 S11 and S12 93

S15 noft((("patient* portal*" or patient*) NEAR/2 portal*) ) 135

S14 noft(((clinical or hospital) NEAR/4 ("decision support system?" or "information system?")) ) 650

S13 noft((meaningful NEAR/1 "use?") ) 406

S12 noft(((computer* or electronic or linkage) NEAR/2 (health or medical) NEAR/2 record?) 2,157

S11 S3 or S4 or S5 or S6 or S7 or S8 or S9 or S10 5,232

S10 noft(("patient reported" NEAR/4 outcome*) ) 342

S9 noft((((body-worn or "body worn" or wearable*) NEAR/2 (biosensor* or sensor* or device? or technolog*)) or (electronic NEAR/1 skin)) ("Self care" OR "self report*" OR "Self monitor*") NEAR/5 (technolog* OR device* OR wearable*)) 9

S8 noft(("Self care" OR "self report*" OR "Self monitor*") NEAR/5 (technolog* or device* or wearable*) ) 243

S7 noft(telemedicine or mHealth or m-health or "mobile health" or ehealth or e-health or "smart technolog*" or smartphone* or "smart phone*" or smartwatch* or "smart watch*") 4,529

S6 noft("quantified self" or lifelog) 43

S5 noft(((personal* or self or patient*) NEAR/2 data* NEAR/2 (tracking or tracke??)) ) 11

S4 noft( ("self recorded health data" or "self-recorded health data" or (("self-recorded" or "self recorded" or "self tracking" or self-tracking or "self tracke??" or self-tracke?? or "self-expressed" or "self expressed" or "personally collected") NEAR/3 (data? or dataset? or "data set?"))) ) 19

Select item 3

S3 S1 OR S2 91

S2 noft(( ((patient* or caregiver*) NEAR/2 (generated or recorded) NEAR/3 (data? or dataset? or "data set?")) ) ) 91

S1 noft(("patient generated data" or "patient generated health data" or "patient-generated data" or "patient-generated health data" or (("patient generated" or "patient-generated") NEAR/3 (data? or dataset? or "data set?"))) ) OR noft(((patient* or caregiver*) NEAR/2 (generated or recorded) NEAR/3 (data? or dataset? or "data set?")) ) 91

**IEEE Xplore Digital Library (ieee.org) August 19, 2019**

1. "patient generated" OR patient-generated

Filters Applied: medical information systems, electronic health records

7 results

1. (("patient generated" OR patient-generated)) AND (electronic health record*)

7 results

1. (("user generated" OR user-generated)) AND (electronic health record*)

3 results

**Inspec (engineeringvillage.com, Elsevier) August 19, 2029**

39 records found in Inspec for 1884-2020:

((("patient generated" or patient-generated or "patient reported" or patient-reported or "user generated" or user-generated or "user reported" or user-reported) AND electronic health record*) WN ALL)
