# Supplementary material for: Patient-generated health data and electronic health record integration: a scoping review
Source: JAMIA Open. 2020 Dec 5;3(4):619–27. doi: 10.1093/jamiaopen/ooaa052 (PMC7969964; doi:10.1093/jamiaopen/ooaa052)
Supplement: ooaa052_Supplementary_Data [file ooaa052_supplementary_data.zip › Supplementary File 2.pdf]

Supplementary File 2

List of Excluded Articles with Reasons for Exclusion

Title	Authors	Year	Journal	Exclusion Reason
Remote collection of questionnaires	Sargious, A.; Lee, S. J.	2014	Clinical and Experimental Rheuma	Wrong study design
Impact of an Electronic Health Record-Integrated Perso	Ryu, B.; Kim, N.; Heo, E.; Y	2017	Journal of Medical Internet Resea	Non-EHR integration
Core Components for a Clinically Integrated mHealth A	Rudin, R. S.; Fanta, C. H.; F	2017	Applied Clinical Informatics	Potential to Integrate Only
Personalized Hypertension Management Using Patient	Lv, N.; Xiao, L.; Simmons, I	2017	Journal of Medical Internet Resea	EHR to App Integration Only
Large-scale clinical implementation of PROMIS comput	Papuga, M. O.; Dasilva, C.;	2018	Health Systems	Wrong setting
Bringing PROMIS to practice: brief and precise sympto	Wagner, L. I.; Schink, J.; Ba	2015	Cancer	Wrong setting
The role of technical advances in the adoption and inte	Jensen, R. E.; Rothrock, N.	2015	Medical Care	Wrong study design
A Pilot Comparison of a Smartphone App With or With	Jamison, R. N.; Jurcik, D. C	2017	Clinical Journal of Pain	Non-EHR integration
Physician perspective on incorporation of oncology pat	Hubbard, J. M.; Grothey, A	2014	Journal of Oncology Practice	Wrong setting
Development of a Smart Mobile Data Module for Fetal	Houze de l'Aulnoit, A.; Bou	2018	Journal of Medical Systems	Potential to Integrate Only
Implementation of Patient-Reported Outcomes Measu	Haskell, A.; Kim, T.	2018	Foot and Ankle International	Not PGHD
The effect of EHR-integrated patient-reported outcom	Harle, C. A.; Marlow, N. M	2016	American Journal of Managed Ca	Not PGHD
Overcoming barriers to implementing patient-reportec	Harle, C. A.; Listhaus, A.; C	2016	Journal of the American Medical I	Wrong study design
Point of Care Research: Integrating patient-generated	Marceglia, S.; D'Antrassi, F	2017	AMIA ... Annual Symposium Proce	Non-EHR integration
EMPOWER-support of patient empowerment by an int	Mantwill, S.; Fiordelli, M.;	2015	BMC Medical Informatics and Dec	Potential to Integrate Only
Integration architecture of a mobile virtual health reco	Hagglund, M.; Scandurra,	2005	Studies in Health Technology and	Not PGHD
eHealth System for Collecting and Utilizing Patient Rep	Girgis, A.; Durcinoska, I.; L	2017	Journal of Medical Internet Resea	Not original article
Study protocol for a controlled trial of an eHealth syste	Girgis, A.; Durcinoska, I.; G	2018	BMC Cancer	Wrong study design
Development and Feasibility Testing of PROMPT-Care,	Girgis, A.; Delaney, G. P.; A	2016	JMIR Research Protocols	Wrong study design
Design and Prestudy Assessment of a Dashboard for Pr	Giordanengo, A.; Arsand, I	2019	JMIR Diabetes	Potential to Integrate Only
Oh, the Places We'll Go: Patient-Reported Outcomes a	Gensheimer, S. G.; Wu, A.	2018	The Patient: Patient-Centered Ou	Not original article
From smartphone to EHR: a case report on integrating	Genes, N.; Violante, S.; Ce	2018	Npj Digital Medicine	Wrong study design
A standardised pre-hospital electronic patient care syst	Gaynor, M.; Myung, D.; Gu	2009	International Journal of Electroni	Not PGHD
Le Bon Samaritain: A Community-Based Care Model Su	Gay, V.; Leijdekkers, P.; Gi	2015	Studies in Health Technology and	Non-EHR integration
Bringing Health and Fitness Data Together for Connect	Gay, V.; Leijdekkers, P.	2015	Journal of Medical Internet Resea	Potential to Integrate Only
Are physicians interested in the quality of life of their p	Fritz, F.; Dugas, M.	2013	Studies in Health Technology and	Wrong setting
Qualitative and quantitative evaluation of EHR-integrat	Fritz, F.; Balhorn, S.; Riek,	2012	International Journal of Medical I	Wrong setting
Framework To Guide The Collection And Use Of Patien	Franklin, P.; Chenok, K.; La	2017	EGEMS	Not original article

Using Technology to Improve Cancer Care: Social Medi	Fisch, M. J.; Chung, A. E.; A	2016	American Society of Clinical Onco	Wrong study design
A mobile feedback system for integrated E-health platf	Fioravanti, A.; Fico, G.; Arr	2011	2011 Annual International Confer	Wrong study design
Patients' acceptance of Internet-based home asthma t	Finkelstein, J.; Hripcsak, G	1998	2012 Annual International Confer	Non-EHR integration
Development of Parkinson Patient Generated Data Col	Kim, D. Y.; Hwang, S. H.; K	2017	Studies in Health Technology and	Non-EHR integration
Healthcare standards based sensory data exchange for	Khan, W. A.; Hussain, M.; .	2012	2012 Annual International Confer	Wrong study design
Electronic Stroke CarePath: Integrated Approach to Str	Katzan, I. L.; Fan, Y.; Speck	2015	Circulation: Cardiovascular Qualit	Not PGHD
The Knowledge Program: an innovative, comprehensiv	Katzan, I.; Speck, M.; Dopl	2011	AMIA ... Annual Symposium Proce	Not PGHD
Remote monitoring of patients with implanted devices	Van der Velde, E. T.; Atsm	2013	European Journal of Preventive C	Potential to Integrate Only
Development of Comprehensive Personal Health Recor	Jung, S. Y.; Kim, J. W.; Hwa	2019	JMIR MHealth and UHealth	Non-EHR integration
Patient-reported outcomes in a large community-base	Juckett, D. A.; Davis, F. N.;	2015	BMC Medical Informatics and Dec	Wrong study design
Congruence of multiple patient-related outcomes with	Johnstone, P. A. S.; Bulls, I	2019	Supportive Care in Cancer	Not PGHD
Value-Based Breast Cancer Care: A Multidisciplinary Ap	Fayanju, O. M.; Mayo, T. L	2016	Annals of Surgical Oncology	Potential to Integrate Only
A mobile health monitoring-and-treatment system bas	El-Sappagh, S.; Ali, F.; Hen	2019	BMC Medical Informatics and Dec	Wrong study design
Integration of Provider, Pharmacy, and Patient-Report	Dixon, B. E.; Alzeer, A. H.;	2016	JMIR Medical Informatics	Potential to Integrate Only
Last Mile Towards Efficient Healthcare Delivery in Swit	Deng, Y.; Burkle, T.; Holm,	2018	Studies in Health Technology and	Non-EHR integration
Interoperability of a mobile health care solution with e	De Toledo, P.; Lalinde, W.,	2006	2006 Annual International Confer	Potential to Integrate Only
A Cloud-Based Virtual Outpatient Clinic for Patient-Cen	de Jong, J. M.; Ogink, P. A.	2018	Journal of Medical Internet Resea	Non-EHR integration
Assessing the feasibility of a mobile health-supported	de Bruin, J. S.; Schuh, C.; S	2018	Artificial Intelligence in Medicine	Non-EHR integration
Twenty-five years of advocacy for patients with gastro	Cutts, T.; Holmes, S.; Keda	2016	BMC Gastroenterology	Non-EHR integration
Palliative Care Planner: A Pilot Study to Evaluate Accep	Cox, C. E.; Jones, D. M.; Re	2018	Annals of the American Thoracic S	Non-EHR integration
Design and testing of Medivate, a mobile app to achiev	Coons, J. C.; Patel, R.; Cole	2019	Journal of the American Pharmac	EHR to App Integration Only
Using technology to improve data capture and integrat	Gurland, B.; Alves-Ferreira	2010	Diseases of the Colon and Rectum	Wrong setting
Automation and Simplification: Drivers of Innovative C	Guattery, J. M.; Johnson, J	2019	Population Health Management	Wrong study design
Visualizing the Patient-Reported Outcomes Measurem	Grossman, L. V.; Mitchell,	2017	AMIA ... Annual Symposium Proce	Wrong study design
Incorporating patient-reported outcome measures into	Griffith, S. D.; Thompson,	2015	Quality of Life Research	Potential to Integrate Only
Overcoming Clinical Inertia: A Randomized Clinical Tria	Greenwood, D. A.; Blozis,	2015	Journal of Medical Internet Resea	Non-EHR integration
Incorporating the patient's voice into electronic health	Chung, A. E.; Basch, E. M.	2015	Journal of the American Medical I	Wrong study design
Hygehos Home: an innovative remote follow-up syster	Carrasco, E.; Sanchez, E.; A	2014	Studies in Health Technology and	Not original article
Going mobile with diabetes support: a randomized stu	Capozza, K.; Woolsey, S.; C	2015	Diabetes Spectrum	Non-EHR integration
Digital health technology and diabetes management	Cahn, A.; Akirov, A.; Raz, I.	2018	Journal of Diabetes	Not original article
A case study of stakeholder perceptions of patient held	Bidmead, E.; Marshall, A.	2016	Digital Health	Potential to Integrate Only
Population-level changes in lifestyle risk factors for car	Benson, G.; Sidebottom, A	2019	Preventive Medicine Reports	Not PGHD
Patient Adoption and Utilization of a Web-Based and M	Bell, K.; Warnick, E.; Nicho	2018	American Journal of Medical Qua	Non-EHR integration
Data Management for Applications of Patient Reported	Bayliss, E. A.; Tabano, H. A	2018	EGEMS	Wrong study design

Supporting clinical practice decisions with real-time pa	Basch, E.; Abernethy, A. P.	2011	Journal of Clinical Oncology	Wrong study design
Wireless remote patient monitoring in older adults	Baig, M. M.; GholamHosse	2013	2013 35th Annual International C	Potential to Integrate Only
Feasibility and accessibility of electronic patient-report	Bae, W. K.; Kwon, J.; Lee, I	2018	Supportive Care in Cancer	Non-EHR integration
Integrating patient-reported outcomes into orthopaed	Ayers, D. C.; Zheng, H.; Fra	2013	Clinical Orthopaedics and Related	Potential to Integrate Only
Developing a real-time electronic symptom monitoring	Avery, K. N. L.; Richards, H	2019	BMC Cancer	Not original article
Providing 'the bigger picture': benefits and feasibility o	Austin, L.; Sharp, C. A.; var	2019	Rheumatology	Potential to Integrate Only
Impact of the Mobile HealthPROMISE Platform on the	Atreja, A.; Khan, S.; Rogers	2015	JMIR Research Protocols	Non-EHR integration
Feasibility test of a UK-scalable electronic system for re	Ashley, L.; Jones, H.; Form	2011	BMC Medical Informatics and Dec	Non-EHR integration
Including Patient-Generated Health Data in Electronic I	Aschettino, L.; Baldwin, K.	2015	Journal of AHIMA	Wrong study design
Outpatient blood pressure monitoring using bi-directio	Anthony, C. A.; Polgreen, I	2015	Journal of the American Society o	Non-EHR integration
Leveraging electronic tablets for general pediatric care	Anand, V.; McKee, S.; Dug	2015	Applied Clinical Informatics	Potential to Integrate Only
Effect of Lifestyle Modification Using a Smartphone Ap	Cho, S. W.; Wee, J. H.; Yoo	2018	Clinical and Experimental Otorhin	Insufficient data
Daily remote peritoneal dialysis monitoring: an adjunct	Chand, D. H.; Bednarz, D.	2008	Peritoneal Dialysis International	Non-EHR integration
Project HealthDesign: a preliminary program-level repo	Casper, G. R.; Brennan, P.	2013	AMIA ... Annual Symposium Proce	Potential to Integrate Only
Tablet-based screening improves continence managem	Beadnall, H. N.; Kuppanda	2015	Annals of Clinical & Translational	Not PGHD
TLC-Asthma: an integrated information system for pati	Adams, W. G.; Fuhlbrigge,	2003	AMIA ... Annual Symposium Proce	Insufficient data
Electronic patient self-Reporting of Adverse-events: Pa	Absolom, K.; Holch, P.; Wa	2017	BMC Cancer	Wrong study design
Management of gastrointestinal symptoms in advance	Abernethy, A. P.; Wheeler	2010	Current Opinion in Supportive & F	Non-EHR integration
Electronic patient-reported data capture as a foundatio	Abernethy, A. P.; Ahmad, .	2010	Medical Care	Non-EHR integration
Enhancing patient engagement and blood pressure ma	Aberger, E. W.; Migliozzi, I	2014	Telemedicine Journal and e-Healt	Non-EHR integration
Electronic patient reported outcomes significantly imp	Shears, A.; Bayman, N.; Ha	2016	Lung Cancer	Wrong study design
Sharing the burden of rheumatoid arthritis through rer	Sharp, C. A.; Austin, L.; Ma	2018	Annals of the Rheumatic Diseases	Insufficient data
582 Electronic patient-reported outcome implementat	Secrest, A. M.; Flint, N. D.;	2019	Journal of Investigative Dermatol	Insufficient data
Leveraging FHIR to Integrate Activity Data with Electro	Saripalle, R. K.	2019	Health and Technology	Potential to Integrate Only
Implementing routine assessment of patient-reported	Mooney, K.; Biber, J.; Hess	2017	Journal of Clinical Oncology	Not original article
Implementation of patient reported outcomes in a bur	McMurtrey, L.; Knitz, D.; V	2018	Journal of Burn Care and Research	Insufficient data
MON-107 TELENEPHROLOGY AND REMOTE PATIENT M	Mansur, A.; Farooqi, M. H	2019	Kidney International Reports	Non-EHR integration
Process and product: Development of a technology en	Mammen, J.; Arcoleo, K. J	2018	American Journal of Respiratory a	Potential to Integrate Only
Integration of data from remote monitoring systems ar	van der Velde, E. T.; Foeke	2012	Netherlands Heart Journal	Potential to Integrate Only
Using smartphones to improve remote monitoring of r	Van Der Veer, S.; Austin, L	2017	Annals of the Rheumatic Diseases	Insufficient data
13Health in diabetes management-the BLink experienc	Van Der Burg, G. J.	2013	Pediatric Diabetes	Insufficient data
The development of E-health tools for the managemer	Van Deen, W. K.; Choi, J. N	2014	Gastroenterology	Insufficient data
Developing and implementing an iPad-based sexual his	Stoner, B.; Schootman, M.	2014	Sexually Transmitted Diseases	Wrong study design
Integrating environmental data into a personal health r	Killeen, J. P.; Chan, T. C.; C	2015	Annals of Emergency Medicine	Insufficient data

Remote patient monitoring system with a focus on ant	Khan, S.; Usmani, A.	2014	BJOG: An International Journal of	Insufficient data
Opal-the oncology patient application	Joseph, A.; Herrera, D.; Kil	2016	Medical Physics	Wrong study design
Connecting Home-Based Self-Monitoring of Blood Pres	Rodriguez, S.; Hwang, K.; \	2019	JMIR Formative Research	Wrong study design
Development of an integrated electronic platform for p	Holch, P.; Warrington, L.; I	2017	Annals of Oncology	Not original article
Mobile health platform for pressure ulcer monitoring w	Rodrigues, J. J.; Pedro, L. N	2013	Health Informatics Journal	Not PGHD
Clinical applications of the ePOCS system: Preliminary f	Wright, P.; Ashley, L.; Craig	2013	Psycho-Oncology	Wrong study design
Automated integration of glucometer data into the ele	Weatherly, J.; Kishnani, S.	2018	Diabetes	Insufficient data
Feasibility of remote, non-invasive, wireless, continuou	Olson, L.; Lexvold, N.; Som	2015	Journal of Cardiac Failure	Insufficient data
The feasibility of utilization of mobile devices to enhan	Ognenovski, V.; Burger, K.	2017	Annals of the Rheumatic Diseases	Non-EHR integration
Use of handheld device to enhance patient reported ou	Nagaraja, V.; Ognenovski,	2018	Arthritis and Rheumatology	Wrong study design
From smartphone to electronic health record (EHR): An	Murali-Ganesh, R.; Tan, Z.	2018	Asia-Pacific Journal of Clinical Onc	Insufficient data
Validation of a scalable efficient interoperable linkage p	Mularski, R. A.; Clark, B.; F	2018	American Journal of Respiratory a	Not PGHD
Systematic clinical application of Patient Reported Out	Hjollund, N. H.; Schougaar	2013	European Journal of Epidemiology	Insufficient data
Development and implementation of a patient-reporte	Deal, C.; Abelson, A.; Calal	2018	Arthritis and Rheumatology	Insufficient data
A prospective trial to evaluate the feasibility of a mobil	Coenen, S.; Weyts, E.; Gee	2018	Journal of Crohn's and Colitis	Insufficient data
Implementation of routine clinical collection of electro	Clark, R. M.; del Carmen, I	2019	Gynecologic Oncology	Not original article
Implementing touch-screen technology to enhance rec	Clark, K.; Matthews, K.; St	2009	Psycho-Oncology	Wrong study design
Harmonization of patient-reported outcomes into EHR	Chung, A.; Stover, A. M.; V	2017	Journal of Clinical Oncology	Not original article
Obtaining patient reported outcome data in the era of	Brister, L. E.; Metheny, L.;	2018	Biology of Blood and Marrow Tran	Insufficient data
Integrating patient-reported outcomes data into the el	Bosch, B.; Hartman, S.; Ca	2018	Journal of Clinical Oncology	Insufficient data
Making my course better: Using patient reported outco	Blackhall, L.; Read, P.; Dav	2014	Journal of Pain and Symptom Mar	Wrong setting
Pilot Study of Home Vitals and Activity Monitoring for A	Bernhard, L.; Coffman, J.;	2019	Biology of Blood and Marrow Tran	Insufficient data
Heart on FHIR: Integrating Patient Generated Data into	Bergquist, T.; Buie, R. W.;	2017	AMIA ... Annual Symposium proce	Potential to Integrate Only
Systematic electronic capture of patient reported outc	Avery, K.; Richards, H.; Po	2019	European Journal of Surgical Onco	Not original article
Feasibility of digital self-report PRO data for monitoring	Avery, K.; Richards, H.; Po	2018	Quality of Life Research	Not original article
Patients' experiences of using a smartphone app for re	Austin, L.; Sanders, C.; Dix	2016	Arthritis and Rheumatology	Not original article
Patients' experiences of using a smartphone app for re	Austin, L.; Sanders, C.; Dix	2017	Rheumatology (United Kingdom)	Non-EHR integration
Patients' experiences of remote monitoring of rheuma	Austin, L.; Sanders, C.; Dix	2017	Annals of the Rheumatic Diseases	Not original article
Online lifestyle tracking only improves weight outcome	Arnold, J.; Tudorascu, D. L	2018	Journal of General Internal Medic	Not original article
Use of electronic patient diaries supports diagnosis of f	Arens-Volland, A.; Feidert,	2011	Clinical and Translational Allergy	Not original article
Refinement and evaluation of a comprehensive disease	Allen, N. A.; Zagarins, S. E.	2012	Diabetes	Not original article
Assistive technology to monitor activity, health and we	Ahanathapillai, V.; Amor, J	2015	Technology and Disability	Non-EHR integration
Toward clinical decision support for chronic pain: Integ	Harle, C.; Schmidt, S.; Fillir	2015	Journal of Pain	Wrong study design
Using technology to facilitate data capture and integra	Gurland, B.; Ferreira, P. C.	2010	Colorectal Disease	Not original article

Using technology to facilitate data capture and integra	Gurland, B.; Ferreira, P.; S	2010	Diseases of the Colon and Rectum	Insufficient data
A fhir-based data flow enabling patients with diabetes	Giordanengo, A.; Bradway	2018	Diabetes Technology and Therape	Potential to Integrate Only
Electronic and paper collection of patient-reported tox	Gilbert, A.; Sebag-Montefi	2016	International Journal of Radiation	Insufficient data
Implementing electronic health recordâ€“integrated s	Garcia, S. F.; Wortman, K.;	2019	Cancer	Not original article
Integrating mobile Health (mHealth) information techr	Galligioni, E.; Caramatti, S	2015	Annals of Oncology	Not PGHD
Using patient-reported and mobile health data in pract	Gadgil, M. D.; Anderson, N	2018	Journal of General Internal Medic	Potential to Integrate Only
Feasibility of utilizing a novel mhealth platform to deliv	Fung, C.; Peckham, J.; Port	2017	Journal of Clinical Oncology	Insufficient data
Feasibility of an electronic implementation method of	Fung, C.; Peckham, J.; Port	2017	Journal of Clinical Oncology	Not original article
Cancer patients' answers to surveys: Incorporation into	French, K. E.; Feeley, T. W	2017	Journal of Clinical Oncology	Insufficient data
Implementation of a treat-to-target quality improveme	Forman, M.; Leatherwood	2018	Arthritis and Rheumatology	Insufficient data
Implementation of electronic patient-reported outcom	Edelen, C.; Spencer, L.	2018	Journal of Pain and Symptom Ma	Insufficient data
Patient-Reported Outcome Measures and Integration I	Pitzen, C.; Larson, J.	2016	Journal of Oncology Practice	Wrong study design
Electronic multidimensional health assessment questic	Pincus, T.	2016	Clinical and Experimental Rheuma	Potential to Integrate Only
Development, usability and acceptability of an integrat	Webers, C.; Beckers, E.; B	2019	RMD Open	Non-EHR integration
Challenges with Patient Adoption of Automated Integr	Weatherly, J.; Kishnani, S.;	2019	Diabetes Technology & Therapeu	Insufficient data
SMART precision cancer medicine: a FHIR-based app tc	Warner, J. L.; Rioth, M. J.;	2016	Journal of the American Medical I	Potential to Integrate Only
Diabetes Educators' Insights Regarding Connecting Mo	Wang, J.; Chu, C. F.; Li, C.;	2018	JMIR MHealth and UHealth	Potential to Integrate Only
Integration of a mobile-integrated therapy with electro	Peeples, M. M.; Iyer, A. K.	2013	Journal of Diabetes Science and T	Wrong study design
Integrating third-party telehealth records with the gen	Paterson, M.; McAulay, A.	2017	Journal of Innovation in Health In	Not original article
Development of an integrated e-health tool for people	Neubeck, L.; Coorey, G.; P	2016	International Journal of Medical I	EHR to App Integration Only
Beyond One-Off Integrations: A Commercial, Substituta	Mandl, Kenneth D.; Gottli	2019	Journal of Medical Internet Resea	Not original article
Device Integration With the EHR: Saving Time, Improvi	Mace, Scott	2016	HealthLeaders Magazine	Not PGHD
The demand for medical devices is growing	Lockner, Julie	2016	Health Management Technology	Wrong study design
Are Physicians Interested in the Quality of Life of their	Fritz, Fleur; Dugas, Martin	2013	Studies in Health Technology and	Wrong setting
NYC hospital prioritizes collection of patient-reported	Castellucci, Maria	2017	Modern Healthcare	Not original article
Preparing for Patient-Generated Documents...Initiative	Brooks Taylor, Lisa	2013	Journal of AHIMA	Wrong study design
Wnats new in "connected" medical devices? Physicians and parents are adopting		2015	Contemporary Pediatrics	Wrong study design
Including Patient-Generated Health Data in Electronic Health Records...Practice G		2015	Journal of AHIMA	Wrong study design
Development of Parkinson Patient Generated Data Col	Dae-Young, Kim; Sun-ho, I	2018	Studies in Health Technology and	Potential to Integrate Only
Integrating data from an online diabetes prevention pr	Mishuris, R. G.; Yoder, J.; \	2016	BMC Medical Informatics and Dec	Wrong study design
Improving Hypertension Control and Patient Engageme	Milani, R. V.; Lavie, C. J.; B	2017	American Journal of Medicine	Insufficient data
Integrating physical activity data with electronic health	Saripalle, R.; Moucek, R.; I	2019	Unknown	Insufficient data
A prototype of mobile app/EHR communication throug	Rossi, E.; Fontelo, P.; Acke	2015	Unknown	Potential to Integrate Only
Attaining role-based, mandatory, and discretionary acc	Rivera SÃ¡nchez, Y. K.; Der	2018	Unknown	Non-EHR integration

E-BRACE: A Secure Electronic Health Record Access Me	Nayak, S.; Hossain, M. A.;	2019	Unknown	Not PGHD
Smart phone based medicine in-take scheduler, remin	Zao, J. K.; Wang, M. Y.; Tsa	2010	Unknown	Not PGHD
The benefits of wireless enabled applications to facilita	Wickramasinghe, N.; Chala	2012	International Journal of E-Health a	Wrong study design
Personalized wearable systems for real-Time ECG class	Walinjkar, A.; Woods, J.; L	2017	Unknown	Wrong study design
The role of Voice Service technologies in creating the n	Vuppalapati, J. S.; Kedari, S	2018	Unknown	Wrong study design
Integrating internet of things and EHealth solutions for	Takpor, T. O.; Atayero, A. A	2015	Unknown	Wrong study design
Review of an electronic health record model to facilitat	Kadambi, V.; Kadambi, N.;	2018	Unknown	Wrong study design
A feasibility study for the integration of a remote patie	Mosmondor, M.; Benc, I.;	2010	Unknown	Wrong study design
Improving user engagement by aggregating and analys	Leijdekkers, P.; Gay, V.; De	2015	Unknown	Potential to Integrate Only
Diabetes Mobile Care: Aggregating and Visualizing Data	Wood, E.; Yang, Q.; Steinb	2019	AMIA Summits on Translational Sc	Non-EHR integration
Incorporating patient generated health data into chror	Danis, C. M.	2015	Unknown	Not original article
Patient Data Integration in Electronic Health Record Sy	Petrova, Galidiya Ivanova	2014	Annual Journal of Electronics	Wrong study design
Hide-n-Sense: Preserving Privacy Efficiently in Wireless	Mare, Shrirang; Sorber, Ja	2014	Mobile Networks & Applications	Potential to Integrate Only
Seamless Integration of ISO/IEEE11073 Personal Healtl	Ignacio, MartÃ-ez; Javier,	2010	Telemedicine & e-Health	Potential to Integrate Only
Sensing Devices and Sensor Signal Processing for Remc	Fanucci, Luca; Saponara, S	2013	IEEE Transactions on Instrumenta	Wrong study design
130: Routine implementation of electronic patient rep	Ala'Aldeen, K.; Stones, N.;	2017	Lung Cancer (01695002)	Non-EHR integration
ePRO: A Maturing and Widely-Preferred Market		2013	Applied Clinical Trials	Not PGHD
Implementation and first results of a tablet-based asse	Schuler, M.; Trautmann, F	2017	Zeitschrift fur Evidenz Fortbildung	Wrong study design
