# Supplementary material for: Patient-generated health data and electronic health record integration: a scoping review
Source: JAMIA Open. 2020 Dec 5;3(4):619–27. doi: 10.1093/jamiaopen/ooaa052 (PMC7969964; doi:10.1093/jamiaopen/ooaa052)
Supplement: ooaa052_Supplementary_Data [file ooaa052_supplementary_data.zip › Supplementary File 3.docx]

Supplementary File 3

Scoping Review Study Characteristics

| Study | Geographic Region | Study Aim | Study Design | Study Setting | Study Population |
| --- | --- | --- | --- | --- | --- |
| Absolom, K., Gibson, A., & Velikova, G. (2019). Engaging patients and clinicians in online reporting of adverse effects during chemotherapy for cancer: The eRAPID System (Electronic Patient Self-Reporting of Adverse Events: Patient Information and aDvice). *Medical Care, 57*, S59–S65. | United Kingdom | To encourage the delivery of timely and appropriate patient-centered clinical advice during cancer treatment using the eRAPID intervention | (No evaluation) | Acute oncology service—dedicated acute-admissions ward and assessments unit | Cancer patients receiving treatment from the National Health Service |
| Ancker, J. S., Mauer, E., Kalish, R. B., Vest, J. R., & Gossey, J. T. (2019). Early adopters of patient-generated health data upload in an electronic patient portal. *Applied Clinical Informatics, 10*(2), 254–260. doi:10.1055/s-0039-1683987 | United States | To describe adoption rates and characteristics of early adopters of PGHD functionality with preliminary data about associations | Observational: Retrospective cohort | Multispecialty faculty practice—physician ambulatory offices and ambulatory hospital-based clinics | 12 providers, 53 patients with any recorded diagnosis of diabetes or gestational diabetes |
| Day, F. C., Pourhomayoun, M., Keeves, D., Lees, A. F., Sarrafzadeh, M., Bell, D., & Pfeffer, M. A. (2019). Feasibility study of an EHR-integrated mobile shared decision making application. *International Journal of Medical* | United States | To test the usability of a trial-tested Web-based patient-education intervention into an EHR-integrated mobile application | Observational: Cross-sectional | Single clinic | 4 providers, 9 patients being screened for prostate specific antigen |
| Supplementary File 2 (Continued) | | | | | |
| Study | Geographic Area | Study Aim | Study Design | Study Setting | Study Population |
| *Informatics, 124*, 24–30. |  |  |  |  |  |
| Fisher, N. D., Fera, L. E., Dunning, J. R., Desai, S., Matta, L., Liquori, V., . . . MacRae, C. A. (2019). Development of an entirely remote, non‐physician led hypertension management program. *Clinical Cardiology, 42*(2), 285–291. | United States | To develop a remote, navigator-led, home-based hypertension program | Experimental: Pre/post | Primary care and specialty clinics | 130 patients with hypertension |
| Girgis, A., Durcinoska, I., Arnold, A., & Delaney, G. P. (2019). Interpreting and acting on the PRO scores from the Patient-reported Outcomes for Personalized Treatment and Care (PROMPT-Care) eHealth system. *Medical Care, 57*, S85–S91. | Australia | To detail methods and processes that informed PROMPT-Care program development | (No evaluation) | Four cancer centers | 400+ patients in 4 cancer centers |
| Gold, H. T., Karia, R. J., Link, A., Lebwohl, R., Zuckerman, J. D., Errico, T. J., . . . Cantor, M. N. (2018). Implementation and early adaptation of patient-reported outcome measures into an electronic health record: A technical report. *Journal of Health and Medical Informatics*. doi:10.1177/1460458218813710 | United States | To describe the design and implementation of creating patient-reported outcomes measures | Mixed methods: Cross-sectional, descriptive | Large urban academic medical center, department of orthopedic surgery | 58 physicians (36,121 visits) |

| Supplementary File 2 (Continued) | | | | | |
| --- | --- | --- | --- | --- | --- |
| Study | Geographical Area | Study Aim | Study Design | Study Setting | Study Population |
| Graetz, I., Anderson, J. N., McKillop, C. N., Stepanski, E. J., Paladino, A. J., & Tillmanns, T. D. (2018). Use of a Web-based app to improve postoperative outcomes for patients receiving gynecological oncology care: A randomized controlled feasibility trial. *Gynecologic Oncology, 150*(2), 311–317. | United States | To evaluate a postoperative Web-based application intervention to provide real-time symptom monitoring to patients who had open bilateral salpingo-oophorectomy surgery | Experimental: Randomized controlled trial | Cancer center | 35 patients diagnosed or with suspected of having gynecological cancer |
| Kumar, R. B., Goren, N. D., Stark, D. E., Wall, D. P., & Longhurst, C. A. (2016). Automated integration of continuous glucose monitor data in the electronic health record using consumer technology. *Journal of the American Medical Informatics Association, 23*(3), 532–537. doi:10.1093/jamia/ocv206 | United States | To pilot and assess the feasibility of automatic integration of continuous glucose monitor data in the EHR using consumer technology | Descriptive | Clinic setting | 1 provider, 10 pediatric patients with insulin-dependent diabetes |
| Leventhal, R. (2015). *How Duke is using HealthKit to get patient-generated data into the EHR*. Retrieved from [https://www.hcinnovationgroup.com/clinical-it/article/13025001/how-duke-is-](https://www.hcinnovationgroup.com/clinical-it/article/13025001/how-duke-is-using-healthkit-to-get-patientgenerated-data-into-the-ehr) | United States | To describe the use of HealthKit to get PGHD into the EHR | (No evaluation) | Outpatient setting | Fewer than 50 patients and providers |

| Supplementary File 2 (Continued) | | | | | |
| --- | --- | --- | --- | --- | --- |
| Study | Geographical Area | Study Aim | Study Design | Study Setting | Study Population |
| using-healthkit-to-get-patient-generated-data-into-the-ehr |  |  |  |  |  |
| Lewinski, A. A., Drake, C., Shaw, R. J., Jackson, G. L., Bosworth, H. B., Oakes, M., . . . Crowley, M. J. (2019). Bridging the integration gap between patient-generated blood glucose data and electronic health records. *Journal of the American Medical Informatics Association, 26*, 667–672. | United States | To examine the feasibility of delivering a telemedicine intervention using processes for integration of PGHD into the EHR | Qualitative | 2 primary care clinics | 35 patients with type 2 diabetes |
| Marquard, J. L., Garber, L., Saver, B., Amster, B., Kelleher, M., & Preusse, P. (2013). Overcoming challenges integrating patient-generated data into the clinical EHR: Lessons from the CONtrolling Disease Using Inexpensive IT–Hypertension in Diabetes (CONDUIT-HID) Project. *International Journal of Medical Informatics, 82*, 903–910. | United States | To remedy technical and procedural challenges before implementing a randomized controlled trial on a low-cost consumer health informatics intervention | Qualitative | Multispecialty medical group | 26 patients |
| Miyamoto, S., Dharmar, M., Fazio, S., Tang-Feldman, Y., & Young, H. M. (2018). mHealth technology and nurse health | United States | To evaluate the impact of a mobile-health-enabled nurse health coaching intervention | Experimental: Randomized controlled trial | Academic health system—primary care clinics | 121 patients with Type-2 diabetes |

| Supplementary File 2 (Continued) | | | | | |
| --- | --- | --- | --- | --- | --- |
| Study | Geographical Area | Study Aim | Study Design | Study Setting | Study Population |
| coaching to improve health in diabetes: protocol for a randomized controlled trial. *JMIR Research Protocols, 7*(2), e45. doi:10.2196/resprot.9168 |  |  |  |  |  |
| Moore, S. L., Fischer, H. H., Steele, A. W., Durfee, M. J., Ginosar, D., Rice-Peterson, C., . . . Davidson, A. J. (2014). A mobile health infrastructure to support underserved patients with chronic disease. *Healthcare, 2*(1), 63–68. | United States | To assess the feasibility of integrating a mobile-health infrastructure with the EMR to support patients with chronic disease | Mixed methods: Prospective cohort, qualitative | Two federally qualified health centers | 135 patients with diabetes |
| Paterson, M., McAulay, A., & McKinstry, B. (2017). Integrating third-party telehealth records with the general practice electronic medical record system: A use case approach. *BMJ Health & Care Informatics, 24*(4), 317–322. | Scotland | To describe a method to produce a report of patient-generated data that is available through their EHR | (No evaluation) | Outpatient setting | 1,200 patients |
| Pennic, J. (2017). *Cedars-Sinai partners with Noteworth to integrate patient-generated data with Epic EMR*. Retrieved from [https://hitconsultant.net/2017/05/16/cedars-sinai-noteworth-](https://hitconsultant.net/2017/05/16/cedars-sinai-noteworth-%20) patient-generated- | United States | To integrate patient-generated data into clinical decision making | (No evaluation) | Outpatient setting | Patients with hypertension, congestive heart failure, diabetes, and thyroid disorders, and maternal–fetal |

| Supplementary File 2 (Continued) | | | | | |
| --- | --- | --- | --- | --- | --- |
| Study | Geographical Area | Study Aim | Study Design | Study Setting | Stuey Population |
| data/#.XgzSqEdKiUk |  |  |  |  | medicine patients who had high-risk pregnancies and related conditions, such as gestational diabetes |
| Sharp, J. (2018). *Effectiveness of patient generated health data in routine clinical care*. Retrieved from <https://www.pchalliance.org/news/effectiveness-patient-generated-health-data-routine-clinical-care> | United States | To demonstrate the flow of data from the patient to the clinician to the researcher. | (No evaluation) | Primary care | Patients with type 2 non-insulin-dependent diabetes |
| Sorondo, B., Allen, A., Bayleran, J., Doore, S., Fathima, S., Sabbagh, I., & Newcomb, L. (2016). Using a patient portal to transmit patient reported health information into the electronic record: Workflow implications and user experience. *eGEMs, 4*(3), Article 12337. doi:10.13063/2327-9214.1237 | United States | To implement an integrated self-report screening tool in a patient portal, to assess workflow and the user experience | Observational | Primary care practices, patient-centered medical home | 24 providers, 72 active care-coordinated, chronic-condition patients |
| Wagner, L. I., Schink, J., Bass, M., Patel, S., Diaz, M. V., Rothrock, N., . . . Rosen, S. (2015). Bringing PROMIS to practice: | United States | To describe a model for implementing PROMIS ePROs into routine cancer care | Mixed methods with three studies:   - Prospective | Outpatient setting | 636 women with gynecological cancer |

| Supplementary File 2 (Continued) | | | | | |
| --- | --- | --- | --- | --- | --- |
| Study | Geographical Area | Study Aim | Study Design | Study Setting | Study Population |
| Brief and precise symptom screening in ambulatory cancer care. *Cancer, 121*, 927–934.\ |  |  | cohort   - Pre/post - Qualitative |  |  |
| Zhang, R., Burgess, E. R., Reddy, M. C., Rothrock, N. E., Bhatt, S., Rasmussen, L. V., . . . Starren, J. B. (2019). Provider perspectives on the integration of patient-reported outcomes in an electronic health record. *JAMIA Open, 2*(1), 73–80. | United States | To examine how well an EHR-integrated patient-reported-outcomes system fits the needs and clinical workflows of different provider groups | Qualitative | Orthopedic and oncology departments | 11 providers |

*Note.* PROMs = patient-reported outcome measures; PGHD = patient-generated health data; EHR = electronic health record.
