# Supplementary material for: Patient-generated health data and electronic health record integration: a scoping review
Source: JAMIA Open. 2020 Dec 5;3(4):619–27. doi: 10.1093/jamiaopen/ooaa052 (PMC7969964; doi:10.1093/jamiaopen/ooaa052)
Supplement: ooaa052_Supplementary_Data [file ooaa052_supplementary_data.zip › Supplementary File 4.docx]

Supplementary File 4

Scoping Review Integration Characteristics

| Study | PGHD Type | EHR Vendor | Mode of Transfer | Developer Platform | Technical Approach |
| --- | --- | --- | --- | --- | --- |
| Absolom, K., Gibson, A., & Velikova, G. (2019). Engaging patients and clinicians in online reporting of adverse effects during chemotherapy for cancer: The eRAPID System (Electronic Patient Self-Reporting of Adverse Events: Patient Information and aDvice). *Medical Care, 57*, S59–S65. | Symptom reports and patient-reported outcomes related to breast, colorectal, and gynecological cancers | Patient Pathway Manager | Active | Qstore and QTool | Web applications interface |
| Ancker, J. S., Mauer, E., Kalish, R. B., Vest, J. R., & Gossey, J. T. (2019). Early adopters of patient-generated health data upload in an electronic patient portal. *Applied Clinical Informatics, 10*(2), 254–260. doi:10.1055/s-0039-1683987 | Blood glucose values, insulin dose, time of insulin administration, free-text notes. | Epic | Active; can upload several values per day. | Apple HealthKit ability was enabled | Not reported. |
| Day, F. C., Pourhomayoun, M., Keeves, D., Lees, A. F., Sarrafzadeh, M., Bell, D., & Pfeffer, M. A. (2019). Feasibility study of an EHR-integrated mobile shared decision making application. *International Journal of Medical Informatics, 124, 24–30.* | 14 data elements: Family history, patient demographics, values, and preferences | Epic | Passive | Not reported. | Seven Epic proprietary non-Fast Healthcare Interoperability Resources Web services |
| Fisher, N. D., Fera, L. E., Dunning, J. R., Desai, S., Matta, L., Liquori, V., . . . MacRae, C. A. (2019). Development of an entirely remote, non‐physician led hypertension management program. *Clinical Cardiology, 42*(2), 285–291. | Average weekly blood pressures | Not reported | Passive | Unspecified digital platform | Bluetooth enabled blood pressure device |

| Supplementary File 3 (Continued) | | | | | |
| --- | --- | --- | --- | --- | --- |
| Study | PGHD Type | EHR Vendor | Mode of Transfer | Developer Platform | Technical Approach |
| Girgis, A., Durcinoska, I., Arnold, A., & Delaney, G. P. (2019). Interpreting and acting on the PRO scores from the Patient-reported Outcomes for Personalized Treatment and Care (PROMPT-Care) eHealth system. *Medical Care, 57*, S85–S91. | Electronic patient-reported outcomes | MOSAIQ | Active | PROMPT-Care | Not reported |
| Gold, H. T., Karia, R. J., Link, A., Lebwohl, R., Zuckerman, J. D., Errico, T. J., . . . Cantor, M. N. (2018). Implementation and early adaptation of patient-reported outcome measures into an electronic health record: A technical report. *Journal of Health and Medical Informatics*. doi:10.1177/1460458218813710 | PROMIS physical function, pain interference, pain intensity measures, and EuroQol 5D | Epic | Active | Northwestern Medicine patient-reported outcomes system | API for PROMIS CAT from Northwestern University |
| Graetz, I., Anderson, J. N., McKillop, C. N., Stepanski, E. J., Paladino, A. J., & Tillmanns, T. D. (2018). Use of a Web-based app to improve postoperative outcomes for patients receiving gynecological oncology care: A randomized controlled feasibility trial. *Gynecologic Oncology, 150*(2), 311–317. | Treatment side effects, physical and emotional symptoms, and functional status | Not reported | Active | Patient Care Monitor platform | Not reported |
| Kumar, R. B., Goren, N. D., Stark, D. E., Wall, D. P., & Longhurst, C. A. (2016). Automated integration of continuous glucose monitor data in the electronic health record using consumer technology. *Journal of the American Medical Informatics Association, 23*(3), 532–537. doi:10.1093/jamia/ocv206 | Glucose values (concentration and trend) obtained by interstitial glucose sensor | Epic | Passive | Apple HealthKit | Publicly available custom Web service, one-time Bluetooth pairing. |

| Supplementary File 3 (Continued) | | | | | |
| --- | --- | --- | --- | --- | --- |
| Study | PGHD Type | EHR Vendor | Mode of Transfer | Developer Platform | Technical Approach |
| Leventhal, R. (2015). *How Duke is using HealthKit to get patient-generated data into the EHR*. Retrieved from [https://www.hcinnovationgroup.com/clinical-it/article/13025001/how-duke-is-using-healthkit-to-get-patientgenerated-data-into-the-ehr](https://www.hcinnovationgroup.com/clinical-it/article/13025001/how-duke-is-using-healthkit-to-get-patientgenerated-data-into-the-ehr%20) | Activity trackers, blood pressure devices, glucose monitoring | Epic | Passive | Apple HealthKit | SMART on Fast Healthcare Interoperability Resources |
| Lewinski, A. A., Drake, C., Shaw, R. J., Jackson, G. L., Bosworth, H. B., Oakes, M., . . . Crowley, M. J. (2019). Bridging the integration gap between patient-generated blood glucose data and electronic health records. *Journal of the American Medical Informatics Association, 26*, 667–672. | Blood glucose values | Epic | Active and passive | Apple HealthKit | Bluetooth |
| Marquard, J. L., Garber, L., Saver, B., Amster, B., Kelleher, M., & Preusse, P. (2013). Overcoming challenges integrating patient-generated data into the clinical EHR: Lessons from the CONtrolling Disease Using Inexpensive IT–Hypertension in Diabetes (CONDUIT-HID) Project. *International Journal of Medical Informatics, 82*, 903–910. | Blood pressure | Epic | Active | Healthvault | Health Level 7 Observation Reporting Interface |
| Miyamoto, S., Dharmar, M., Fazio, S., Tang-Feldman, Y., & Young, H. M. (2018). mHealth technology and nurse health coaching to improve health in diabetes: protocol for a randomized controlled trial. *JMIR Research Protocols, 7*(2), e45. | Physical activity, sleep, and nutrition data | Epic | Passive | Apple HealthKit | Not reported |

| Supplementary File 3 (Continued) | | | | | |
| --- | --- | --- | --- | --- | --- |
| Study | PGHD Type | EHR Vendor | Mode of Transfer | Developer Platform | Technical Approach |
| doi:10.2196/resprot.9168 |  |  |  |  |  |
| Moore, S. L., Fischer, H. H., Steele, A. W., Durfee, M. J., Ginosar, D., Rice-Peterson, C., . . . Davidson, A. J. (2014). A mobile health infrastructure to support underserved patients with chronic disease. *Healthcare, 2*(1), 63–68. | Blood sugar, step counts, blood pressure | Not reported | Active | Patient Relationship Management Software Platform | Not reported |
| Paterson, M., McAulay, A., & McKinstry, B. (2017). Integrating third-party telehealth records with the general practice electronic medical record system: A use case approach. *BMJ Health & Care Informatics, 24*(4), 317–322. | Blood pressure measurements | Not reported | Active | Intersystems Ensemble | Indicated no API available |
| Pennic, J. (2017). *Cedars-Sinai partners with Noteworth to integrate patient-generated data with Epic EMR*. Retrieved from [https://hitconsultant.net/2017/05/16/cedars-sinai-noteworth-](https://hitconsultant.net/2017/05/16/cedars-sinai-noteworth-%20) Patient-generated-data/#.XgzSqEdKiUk | Blood pressure, blood glucose level, weight, etc., and behavioral data, medication adherence, mood, activity, etc. | Epic | Passive | Not reported | Not reported |
| Sharp, J. (2018). *Effectiveness of patient generated health data in routine clinical care*. Retrieved from <https://www.pchalliance.org/news/effectiveness-patient-generated-health-data-routine-clinical-care> | Blood glucose values | Epic | Passive | Validic | Bluetooth-enabled glucometer |

| Supplementary File 3 (Continued) | | | | | |
| --- | --- | --- | --- | --- | --- |
| Study | PGHD Type | EHR Vendor | Mode of Transfer | Developer Platform | Technical Approach |
| Sorondo, B., Allen, A., Bayleran, J., Doore, S., Fathima, S., Sabbagh, I., & Newcomb, L. (2016). Using a patient portal to transmit patient reported health information into the electronic record: Workflow implications and user experience. *eGEMs, 4*(3), Article 12337. doi:10.13063/2327-9214.1237 | Wellness questionnaire survey | GE Centricity | Active | Not reported | Not reported |
| Wagner, L. I., Schink, J., Bass, M., Patel, S., Diaz, M. V., Rothrock, N., . . . Rosen, S. (2015). Bringing PROMIS to practice: Brief and precise symptom screening in ambulatory cancer care. *Cancer, 121*, 927–934.\ | Survey questions: Fatigue, pain interference, physical function, depression, and anxiety | Epic | Active | Not reported | Health Level 7 |
| Zhang, R., Burgess, E. R., Reddy, M. C., Rothrock, N. E., Bhatt, S., Rasmussen, L. V., . . . Starren, J. B. (2019). Provider perspectives on the integration of patient-reported outcomes in an electronic health record. *JAMIA Open, 2*(1), 73–80. | PROMIS CATs survey questions: Pain interference, physical function, social function, pain-intensity short form, fatigue, depression, anxiety | Epic | Active | Northwestern Medicine patient-reported outcomes system | Not reported |

*Note.* PGHD = patient-generated health data; EHR = electronic health record; API = application programming interface; SMART = substitutable medical applications and reusable technologies.
